# Exercise and Skeletal Muscle‐Derived Extracellular Vesicles: Their Cargo, Release, and Role in Metabolic Regulation

**DOI:** 10.1002/jex2.70139

**Published:** 2026-05-09

**Authors:** Jeremy Boulestreau, Scott K. Ferguson, Luke Nelson, Noemi Polgar

**Affiliations:** ^1^ Department of Anatomy, Biochemistry, and Physiology John A. Burns School of Medicine, University of Hawaii Honolulu Hawaiʻi USA; ^2^ Department of Human Factors and Behavioral Neurobiology, Integrative Aerospace and Exercise Physiology Laboratory Embry‐Riddle Aeronautical University Daytona Beach Florida USA

**Keywords:** exercise, extracellular vesicle, metabolic disorders, skeletal muscle, T2DM

## Abstract

The health benefits of exercise are well established in the prevention and management of metabolic disorders such as obesity and diabetes. Emerging evidence indicates that extracellular vesicles (EVs) may contribute to the beneficial effects of exercise. These membrane‐bound nanoparticles carry bioactive molecules, such as proteins, lipids, and RNAs, and facilitate intercellular communication. Of note, exercise has been shown to significantly influence EV release and cargo, enhancing their ability to mediate metabolic benefits across various tissue types. Recent investigations have demonstrated that skeletal muscle‐derived EVs (SkM‐EVs) can improve insulin sensitivity, promote glucose homeostasis, and modulate lipid metabolism in recipient cells and tissues. Additionally, exercise‐induced SkM‐EVs are enriched in specific proteins and microRNAs that activate key signaling pathways essential for glucose homeostasis, thereby providing protective effects against insulin resistance, inflammation, and other hallmarks of metabolic dysfunction. In this review, we summarise the current understanding of the effects of exercise on SkM‐EV release, molecular cargo, and the potential mechanisms by which they exert metabolic benefits. By doing so, we highlight the potential of SkM‐EVs as therapeutic tools for treating diabetes and related metabolic disorders.

## 1 Introduction

Regular exercise significantly reduces morbidity associated with metabolic disorders, including type 2 diabetes, thereby extending the health span (e.g., the portion of life spent free from chronic disease, disability, and functional decline) (Gaesser et al. 2025). This is critically important given that the health span is the period in which people can sustain adequate physical and cognitive function to engage in activities of daily living that define life quality (Seals and Melov 2014). In this regard, engaging in physical activity promotes numerous favorable physiological adaptations with both short and long‐term benefits. For instance, a bout of acute exercise increases glucose uptake through insulin‐independent mechanisms and improves insulin sensitivity in major metabolic tissues for up to 48 h post‐exercise (Macpherson et al. 2015; Trefts et al. 2015; Sjøberg et al. 2017). The short‐term gains in metabolic function accumulate over time, as long‐term exercise leads to sustained improvements in cardiorespiratory fitness, increased muscular strength, and better blood pressure regulation (Piercy et al. 2018), each of which is strongly correlated with reduced morbidity and mortality (Lang et al. 2024). These adaptations further support the value of exercise as a cornerstone lifestyle intervention for individuals with type 2 diabetes, as it improves glycemic control and insulin sensitivity by reducing adiposity, increasing muscle mass, enhancing mitochondrial biogenesis, and activating insulin‐independent pathways of skeletal muscle glucose uptake. All of the aforementioned physiological changes contribute to preserving metabolic health and extending the health span (Chow et al. 2022).

On a molecular level, the changes induced by regular physical activity that play a critical role in initiating and maintaining the processes needed for a healthy metabolic profile are complex. Therefore, the precise mechanisms underlying these beneficial effects of exercise remain incompletely understood. However, gaining insight into the molecular mechanisms by which regular exercise supports healthy metabolic control and extends health span in adults is essential for advancing public health initiatives. A growing body of evidence highlights the contribution of exercise‐induced signaling factors, collectively termed *exerkines*, to improved metabolic control *in vivo*. Exerkines are released in response to acute and chronic physical activity and exert their effects through endocrine, paracrine, and autocrine pathways. Exerkines also originate from various organs, cells, and tissues, especially skeletal muscle. These factors have shown promise in promoting metabolic health, and they hold potential therapeutic applications for enhancing the effects of exercise to treat not only type 2 diabetes mellitus but also obesity as well (Chow et al. 2022).

Among the signaling molecules released during exercise, peptides, cytokines, mRNAs, and microRNAs (miRNAs) have received increasing attention. Of note, many of these molecules are packaged within extracellular vesicles (EVs). These EVs are lipid‐bound nanoparticles released by nearly all cell types into the extracellular space, serving as mediators of intercellular communication (Welsh et al. 2024). These vesicles can deliver their cargo to both local and distant tissues, allowing them to contribute to the systemic benefits of exercise. Skeletal muscle, the primary tissue activated during physical activities, appears to be a key source of these EVs, supporting the notion of a complex interplay between muscle activity and systemic health (Guescini et al. 2015; Estrada et al. 2022; Mastrototaro and Roden 2024). Challenges in standardizing exercise protocols and EV isolation, as well as EV characterization methods, have hindered the consistent observation of EV‐mediated benefits across models. Expanding the area of research provides a framework for exploring the multifaceted benefits of exercise and its potential applications in disease prevention and treatment. But a deeper understanding of SkM‐EV biogenesis, cargo composition, and downstream effects will be critical for harnessing their potential to improve metabolic control and delay the onset of metabolic and age‐related diseases. By exploring the changes in EV biogenesis and cargo content in the context of systemic responses to physical activity, this review highlights the emerging role of SkM‐EVs, particularly small EVs (i.e., those <200 nm in diameter), in mediating the beneficial effects of exercise on metabolism by focusing on peer‐reviewed studies published between 2015 and 2026, with earlier landmark studies included when relevant. Electronic databases, PubMed and Google Scholar, were used to search for relevant literature up to January 2026, using the search words: ‘exercise’, ‘EVs’, ‘exosomes’, ‘microvesicles’, ‘microparticles’, ’muscle’, and ‘skeletal muscle’. Original works, as well as review articles and their secondary references, published since 2015 were reviewed to evaluate their relevance to our topic of interest. The key search terms used were: ‘exercise’, ‘physical exercise’, ‘EVs’, ‘exosomes’, ‘microvesicles’, and ‘microparticles’. We focused on the effects of exercise on metabolism; therefore, ‘metabolism’ was also included as a key search term. We also performed searches evaluating bioactive miRNA and their pathways.

This review highlights that, while numerous studies show changes in circulating EVs following exercise, the specific contribution of skeletal muscle‐derived EVs remains mostly unresolved.

## 2 How Exercise Impacts Circulating EVs

### 2.1 Exercise Intensity, Duration, and Modality Affect Circulating EV Concentrations

Exercise triggers the release of EVs, including exosomes (a small EV species), into the circulation. These EVs originate from various tissues and may contribute to systemic communication and adaptive mechanisms during and after exercise (Frühbeis et al. 2015; Whitham et al. 2018; Annibalini et al. 2019). However, findings regarding circulating EV concentrations following a bout of exercise are inconsistent (J. Lovett et al. 2024), with some investigations showing significant increases in circulating EV concentrations (Oliveira et al. 2018; Bei et al. 2017; Dimassi et al. 2018), while others show no change (Burke et al. 2024; Maggio et al. 2023) or even a decrease (Rigamonti et al. 2020). In light of these inconsistencies, we have to consider the complex nature of EV release (and uptake) dynamics, as these discrepancies may stem from methodological variation, particularly the timing of blood collection relative to exercise, which can substantially impact the quantity of circulating EVs (Nederveen et al. 2021; Llorente et al. 2024).

Emerging evidence suggests that EV release is also dependent on exercise intensity (C. Ma et al. 2018), with a dose‐related increase in EVs observed following higher‐intensity training (Oliveira et al. 2018). High‐intensity exercise has been generally linked with a transient spike in EV levels (Frühbeis et al. 2015; Whitham et al. 2018), although this trend has been challenged by a recent publication reporting decreased particle counts (X. Zhang et al. 2026). However, the effects of moderate‐intensity exercise and resistance training are more variable (Dimassi et al. 2018; C. Ma et al. 2018; Oliveira et al. 2018; Just et al. 2020; J. A. C. Lovett et al. 2018; Rigamonti et al. 2020; X. Zhang et al. 2026). This suggests that the modality, intensity, and duration of the exercise significantly affect the overall EV release and their clearance from the circulation (see review (Llorente et al. 2024)). Interestingly, the EV‐release in response to exercise is also influenced by factors such as sex, the presence of metabolic disease, and age (Conkright et al. 2022; Kargl et al. 2024; Burke et al. 2024; Xhuti et al. 2023; Warnier et al. 2022). For example, one study found that acute exercise increased circulating EVs after 12 weeks of training in healthy men but not in women (Kargl et al. 2024). In contrast, another study showed that, while pre‐ and post‐exercise EV concentrations were not significantly different between sexes, women demonstrated higher concentrations of circulating muscle‐derived EVs (as defined by the presence of the alpha sarcoglycan marker) compared to men following an acute bout of resistance exercise (Conkright et al. 2022). These studies compared the effects of acute exercise between trained and non‐trained subjects, respectively, so the possibility of different sex‐based adaptations to exercise at the root of this contradiction cannot be excluded. A factor potentially contributing to sex‐based differences in EV release could be differences in muscle fiber‐type composition, as women tend to have a higher proportion of oxidative type I fibers than men (Nuzzo 2024), and highly oxidative muscle tissue has been shown to release more EVs than glycolytic tissue (Estrada et al. 2022; Kargl et al. 2023, 2024).

**FIGURE 1:**
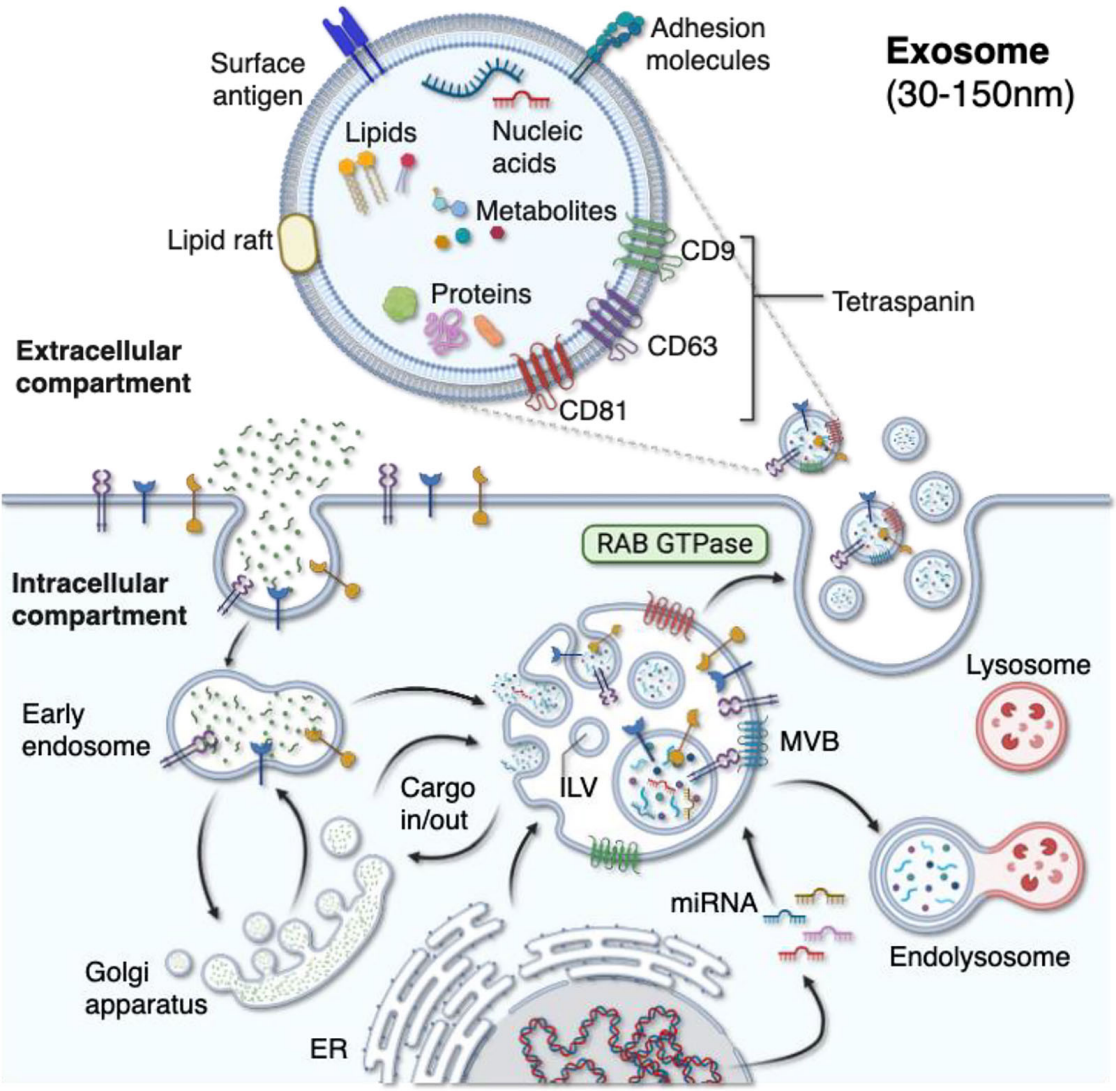
**Overview of exosome biogenesis**. Exosomes are nano‐sized lipid‐bound vesicles whose biogenesis occurs through multivesicular bodies (MVBs). MVBs originate from the endocytic pathway, where inward budding of the endosomal membrane leads to the formation of MVBs. These MVBs can interact with other organelles, including the trans‐Golgi network (TGN) and endoplasmic reticulum (ER), enabling the sorting of diverse molecules, including proteins, RNAs, metabolites or lipids. After the maturation of MVBs, they can either fuse with the lysosome to be degraded or fuse with the plasma membrane to release intraluminal vesicles (ILVs), the precursors of the so‐called exosomes.

In addition to exercise intensity, body composition and other metabolic disruptions might also influence circulating EV concentrations (Amosse et al. 2018). For instance, in one study, men with higher body fat percentages (>30%) exhibited increased EV concentrations both at baseline and after resistance exercise training compared to those with lower body fat percentages (a difference not seen among women (Burke et al. 2024)). Conversely, others reported that healthy men with a normal body mass index (BMI) of 23.5 ± 0.5 kg·m^−2^, showed a transient rise in EV concentrations during an acute bout of exercise, accompanied by elevated expression of EV markers (TSG101 and CD81), which returned to baseline within 30 min post‐exercise. In contrast, in the same study, the prediabetic group of individuals with higher BMIs of 27.3 ± 1.2 kg·m^−2^ exhibited no significant changes in EV concentrations or EV marker protein levels in response to exercise. In this prediabetic group, exercise resulted in an upregulation in skeletal muscle transcripts related to multivesicular body (MVB), an intracellular endosomal organelle serving as a key site of EV formation, such as TSG101, ALIX and CD9 (Figure 1). However, these changes in transcript abundance were not reflected at the protein level. This may indicate either a true lack of effect on EV formation, a delayed protein response, or increased EV release within the experimental timeframe (Warnier et al. 2022). Altogether, interpreting how metabolic state modulates exercise‐induced EV responses is complicated by heterogeneity in study designs, including variations in exercise type, EV isolation method and timing of sample collection.

While exercise intensity, sex, and body composition all seem to impact EV release (and some evidence points to the differential release of EV subtypes in response to physical activity), it is not clear if this activity would have a targeted effect on specific EV species. Furthermore, unlike EV concentration, EV size does not appear to be influenced by exercise (Frühbeis et al. 2015; Oliveira et al. 2018; Whitham et al. 2018; Annibalini et al. 2019; Brahmer et al. 2019; Just et al. 2020; Kawanishi et al. 2023; Xhuti et al. 2023; Kargl et al. 2024; J. Lovett et al. 2024; Burke et al. 2024), as only one study showed a significant EV mean size decrease by only 3% in men following resistance exercise, but there was no significant difference observed in women (Conkright et al. 2022).

### 2.2 Exercise Modifies Circulating EV Cargo

The significance of SkM‐EVs signaling relative to other EV sources is still unclear. Understanding the tissue origins of post‐exercise circulating EVs and defining their cargo is therefore crucial for deciphering their role in multisystemic crosstalk responsible for mediating the beneficial effects of exercise. Likely due to the relative ease of sample collection, the majority of studies focus on circulating, plasma‐derived EVs, representing a mixed population of EVs originating from various tissues. However, the lack of reliable skeletal muscle‐specific EV markers makes determining the origin of the circulating EVs a challenge, creating further uncertainty as to the tissue‐specific effects of exercise on EV release and the associated signals.

#### 2.2.1 miRNA Cargo of Circulating EVs

Despite the challenges associated with collecting EVs, we have gained some information about their cargo through the use of multi‐omics approaches. EVs released during exercise are known to carry various RNA types, including miRNAs (Oliveira et al. 2018; Malvandi et al. 2024; Fernandez‐Sanjurjo et al. 2024; Burke et al. 2024; Just et al. 2020; Castaño et al. 2020; D'Souza et al. 2018; Katayama et al. 2025). To date, only one study has investigated mRNA content in post‐exercise circulating EVs, reporting exercise‐associated changes in angiogenesis and immune regulation (Kawanishi et al. 2023). While several studies have examined exercise‐induced EVs, none have specifically addressed the mRNA cargo of circulating EVs in the context of the metabolic benefits of exercise. Accordingly, we chose to focus this review primarily on miRNAs, and particularly within SkM‐EVs, to reflect the current state of the literature and the availability of data. miRNAs packaged within EVs represent only a subset of cell‐free plasma miRNAs, yet they appear to be particularly responsive to metabolic challenges like exercise. For example, in 2015, the first study investigating the effect of exercise on the miRNA content of circulating EVs reported a significant increase in miR‐181a‐5p (Guescini et al. 2015), a miRNA involved in the regulation of hepatic glucose and lipid metabolism (Du et al. 2017). Subsequently, a study involving high‐intensity interval cycling in young men revealed that circulatory EVs' miRNA levels rose ∼50‐fold post‐exercise (D'Souza et al. 2018). Remarkably, this rise was not reflected in the overall miRNA abundance in muscle tissue or plasma, highlighting a marked discrepancy of miRNA content among EV, muscle, and plasma samples. This suggests that exercise induces either an increase in specific miRNA‐containing small EVs or a selective enrichment of miRNAs within these vesicles. Accordingly, post‐exercise circulating EVs carry a distinct miRNA signature, as supported by the identification of miRNAs present both in skeletal muscle and small circulating EVs but absent in plasma, suggesting that a subset of these EVs may originate from skeletal muscle (D'Souza et al. 2018). This unique miRNA signature may serve as a guide to help identify key mediators of inter‐organ communication in response to exercise.

The current literature highlights that, depending on the exercise modality investigated, different miRNAs are selectively enriched as cargo. Of note, specific miRNAs that are crucial in exercise‐induced cellular responses were found differentially expressed after acute aerobic exercise (AAE) (rno‐miR‐330‐5p, 10b–5p, 142–3p, 410–3p were upregulated, and rno‐miR‐128‐3p, 103–3p, 148a–3p, 191a–5p, 93–5p, 25–3p, 142–5p, 3068–3p were downregulated). These miRNAs were predicted to target and inhibit the MAPK pathway (Oliveira et al. 2018). As the MAPK signaling pathway plays a critical role in regulating cellular stress responses, muscle adaptation, and mitochondrial biogenesis, the miRNA changes can influence the exercise response regulated by the MAPK pathway, and consequently, the overall health span. Longer‐term aerobic training, on the other hand, appears to impact regulators of glucose and lipid metabolism, including miR‐21 (Liu et al. 2022), miR‐146a (Li et al. 2020), and miR‐150 (Ju et al. 2020; Kang et al. 2018), as these miRNAs were increased following eight weeks of training in studies on human EVs (Dimassi et al. 2018). Supporting the role of EV‐derived miRNAs impacting fuel metabolism, a recent study conducted in healthy young individuals reported a significant increase of miR‐136‐3p (targeting the nardilysin convertase (NRDC) gene) in circulating EVs following three weeks of endurance (aerobic) exercise training. When miR‐136‐3p was transfected into human myotubes, it increased glucose uptake, induced a glycolytic shift, and led to elevated basal and ATP‐linked mitochondrial respiration as quantified by a Seahorse XFe24 extracellular flux analyzer by detecting extracellular acidification and oxygen consumption rates, respectively (Katayama et al. 2025). Similar to aerobic exercise, resistance training is also associated with an acute rise in circulating EVs, and as a consequence, a rise in EV‐encapsulated miRNAs, like miR‐206 and miR‐146a. Of these factors, the muscle‐specific ‘myomiR’, miR‐206, plays a key role in muscle development and differentiation. Conversely, miR‐146a is a key regulator of glucose metabolism, particularly in the liver, and its dysregulation is implicated in metabolic disorders like type 2 diabetes and insulin resistance (Li et al. 2020; Roos et al. 2021), both of which lead to increased morbidity and mortality. The post‐exercise increase of miR‐206 and miR‐146a suggests that muscle contributes at least in part to the higher levels of circulating EVs following a bout of exercise (Annibalini et al. 2019).

Supporting these observations, a resistance training model using electrical pulse stimulation (EPS) in mice revealed an increase in circulating EVs enriched with muscle‐specific miR‐206, as well as miR‐1, miR‐133a and miR‐133b. This was accompanied by changes in mRNA expression associated with angiogenesis and immune regulation, suggesting that EVs may mediate key molecular adaptations to resistance exercise (Kawanishi et al. 2023).

Not only resistance training but high‐intensity interval training (HIIT) (an exercise modality involving brief bouts of intense activity followed by periods of recovery) alters the miRNA profile of circulating EVs in mice, increasing levels of myomiRs such as miR‐133a, miR‐133b targeting insulin‐regulated transcription factor forkhead box O1 (FoxO1) (Castaño et al. 2020). These miRNAs regulate muscle physiology, myogenesis, glucose, and lipid metabolism, all of which are crucial for adaptive responses to exercise (Malvandi et al. 2024).

Exercise not only elevates the abundance of circulating EV‐associated miRNAs but can also suppress specific species that may negatively impact metabolic health. For example, Castaño et al. 2020 reported that HIIT in mice led to a downregulation of miR‐192, an exosomal miRNA often elevated in obesity and associated with metabolic dysfunction (Castaño et al. 2018). Similarly, blood flow‐restricted resistance exercise (BFRE), which involves partial vascular occlusion during resistance training, significantly altered the circulating EV miRNA profile. BFRE resulted in upregulation of miRNAs such as miR‐182‐5p, miR‐1294, and let‐7b‐5p, while concurrently suppressing others, including miR‐19b‐3p, miR‐221‐3p, and miR‐21‐5p. Many of these miRNAs target genes involved in muscle regeneration and metabolic regulation, such as Cyclin D1, IGF‐1R, FoxO3, and p53. Enrichment analysis linked these targets to key signaling pathways regulating muscle adaptation, including mTOR, AKT/mTOR, TGFβ, MAPK, and FoxO signaling. Of note, EVs collected after BFRE were shown to promote muscle precursor cell proliferation and therefore suggest a role in muscle repair and regeneration (Just et al. 2020).

The above results highlight the need for studies designed to compare different exercise modalities directly. Recent work has partially filled this gap by examining EV quantity and miRNAs in response to various exercise regimens, including AAE, aerobic training (AT), acute maximal aerobic exercise (AMAE), and altitude aerobic training (AAT). While circulating EV levels appeared unchanged across all exercise modalities, distinct patterns of miRNA regulation were observed. miR‐206 and miR‐133b were upregulated following AAE and AMAE, whereas miR‐486‐5p increased only after AMAE. Interestingly, the level of these miRNAs remained stable after AAT, suggesting modality‐dependent regulation of EV miRNA cargo (Maggio et al. 2023). A separate case‐control study of 16 sedentary men, 16 Olympic endurance athletes, and 16 resistance athletes found lower levels of miR‐16‐5p, miR‐19a‐3p, and miR‐451a in circulating EVs of athletes, with miR‐25‐3p specifically lower in endurance athletes, suggesting a role for the EV‐transported miRNAs in exercise adaptation and energy metabolism (Fernandez‐Sanjurjo et al. 2024). Collectively, these findings emphasize that exercise influences the miRNA cargo of EVs, with specific miRNAs being differentially regulated depending on the exercise modality. In addition, the EV‐miRNA response to exercise is not only sensitive to training status and modality but also contributes to key physiological processes, including muscle adaptation, metabolism, and inflammation via regulation of signaling pathways such as MAPK, mTOR, and FoxO. The distinct miRNA profiles observed across different training types underscore the dynamically regulated role of EVs in mediating inter‐tissue communication and exercise‐induced adaptations.

#### 2.2.2 Protein Cargo of Circulating EVs

EV release during exercise is not limited to a single cell type; rather, it involves multiple tissues, thus facilitating interorgan communication (Nederveen et al. 2021). Besides miRNAs, exercise also stimulates the release of proteins as EV cargo, and the specific cargo composition of EVs often reflects their parental cell of origin. Of note, circulating EV surface biomarker profiles correlate with insulin sensitivity in humans, while high‐intensity aerobic exercise increases several insulin action‐related EV subpopulations, including skeletal muscle‐associated EVs. These observations support circulating EVs as biomarkers of metabolic health (X. Zhang et al. 2026). Understanding the origin and subtypes of these EVs is essential for clarifying their roles in mediating the beneficial effects of exercise.

Just et al. 2020 have identified five EV markers significantly altered after exercise: integrin alpha 2b (ITGA2B/CD41), neural cell adhesion molecule (NCAM), interleukin‐2 receptor alpha chain (IL2RA/CD25), and programmed cell death 6 interacting protein (PDCD6IP/ALIX). ITGA2B/CD41 is primarily expressed in hematopoietic cells, while NCAM is found in brain and peripheral neuronal cells, glial cells, NK cells, and endothelial cells. Interestingly, muscle stem cells also express this marker abundantly, likely in order to regulate interactions between neurons and muscle (Capkovic et al. 2008). IL2RA/CD25 is associated with the bone marrow and immune system, and PDCD6IP/ALIX is ubiquitously expressed and plays essential roles in EV biogenesis, including formation, secretion, sorting, and miRNA packaging (Iavello et al. 2016). Notably, their relative abundance is increased in the circulating EVs following BFRE, indicating a potential link between exercise, blood flow, and EV‐mediated cellular communication. Conversely, the same study reported a significant decrease in EVs carrying the flotillin‐1 surface marker (Just et al. 2020), highlighting the selective regulation of EV subpopulations by exercise and the need to further analyze and understand these responses.

In accord, a study using EV Array and flow cytometry on circulating EVs collected from athletes post‐exercise identified EVs from various cell types, including endothelial cells, as well as leukocytes and other immune cells (Brahmer et al. 2019). Supporting the idea that EVs from diverse cell types contribute to exercise‐induced signaling, in a study of 17 young men, proteomic analysis identified 558 proteins in circulating EVs following high‐intensity exercise. Among these EV cargo proteins, many originated from tissues such as skeletal muscle, liver, and adipose tissue (Kobayashi et al. 2021).

Another proteomic analysis provided compelling evidence for the contribution of EVs to the coordinated responses to exercise. Here, following a one‐hour bout of cycling in healthy individuals, more than 300 proteins were found to be increased in the EVs, with a significant enrichment of several classes of proteins associated with exosomes and small vesicles. Notably, several glycolytic enzymes were significantly increased in the exercise samples compared with the rest and recovery samples (Whitham et al. 2018). These findings suggest that exercise not only alters the circulating EV proteome but may also utilize EVs as mediators of systemic metabolic adaptation.

The key underlying feature of the above investigations is that EVs are released from a wide variety of tissues. The variability in EV release in terms of the tissue of origin and cargo content underscores the complexity of exercise‐related signaling. Future research should focus on identifying the specific tissues contributing to circulating EVs post‐exercise and characterizing their distinct molecular cargo to clarify their role in mediating exercise‐induced adaptations. A central finding from these investigations is that EVs are dynamically released by many different tissues and that variation in both their tissue of origin and subpopulations highlights the complexity of exercise response. Identification of the specific tissues that contribute to circulating EVs and characterization of their distinct molecular cargo would advance our understanding by clarifying the roles of EVs in mediating exercise‐induced adaptations.

### 2.3 Tissue Targeting and Downstream Effects of Exercise‐Induced Circulating EVs

Understanding how the post‐exercise circulating EV population contributes to metabolic regulation not only requires identifying their cargo but also identifying their target tissues and the various signaling processes they influence. The hypothesis that EVs facilitate tissue crosstalk during exercise is grounded in their uptake by recipient tissues. Whitham et al. evaluated the biodistribution of circulating EVs by first isolating EVs from exercised and sedentary mice and, following fluorescent labeling, injecting them intravenously into recipient animals (Whitham et al. 2018). Three hours post‐injection, fluorescence was observed in the abdominal viscera of mice that received vesicles from exercised donors but not from sedentary ones. In follow‐up experiments, fluorescence was restricted to the liver, with significantly higher uptake in mice receiving EVs from exercised donors but not rested ones, indicating enhanced liver localization of exercise‐induced EVs. It is thought that small EVs target specific tissues via surface adhesion proteins such as integrins and tetraspanins. Notably, exercise induces a marked increase in several adhesion proteins in the bloodstream, many of which are transferred to liver cells through EVs from exercised mice. This suggests that exercise‐driven changes in adhesion proteins likely regulate the tissue‐specific targeting of vesicles, particularly to the liver (Whitham et al. 2018). In agreement with these findings, EVs circulating in the plasma of high‐intensity interval training‐induced mice, when isolated and injected intravenously into sedentary mice, were shown to accumulate in the liver and skeletal muscle six hours after injection (Castaño et al. 2020).

But circulating EVs do not only target the liver or skeletal muscle cells. A recent study reported that following an acute bout of resistance exercise, circulating EVs transferred their myomiR cargo, miR‐1, to adipose tissue. The miR‐1 target genes identified were caveolin 2 (CAV2) and tripartite motif‐containing 6 (TRIM6), and the overexpression of miR‐1 in differentiated human adipocyte‐derived stem cells led to downregulation of these target genes and enhanced catecholamine‐induced lipolysis. These findings highlight a potential EV‐mediated mechanism through which circulating EVs communicate with adipose tissue, modulating lipolysis (Burke et al. 2024).

Once produced, EVs travel to the target tissues, where they will release their cargo, inducing physiological changes in the organism and, often specifically, in the target organ. Post‐exercise EVs enriched from the plasma of exercise‐induced mice, when delivered into sedentary animals, enhanced glucose tolerance, improved insulin sensitivity, and reduced plasma triglyceride levels, affecting whole‐body metabolism (Castaño et al. 2020). EVs isolated following BFRE, on the other hand, were found to specifically activate and induce S‐phase entry in human primary muscle precursor cells, whereas EVs isolated before BFRE did not influence proliferation (Just et al. 2020). These results support the idea that exercise stimulates EV release to facilitate muscle remodeling and growth as well.

Exercise‐induced circulating EVs have been shown to contain several glycolytic enzymes (Whitham et al. 2018), highlighting their potential role in supporting the heightened energy demands associated with exercise. This finding is consistent with prior evidence that EVs can increase glycolytic activity in recipient cells (Garcia et al. 2016). Further insights come from a proteomic analysis of circulating EVs in a cohort of 17 young men following high‐intensity exercise, which revealed 20 proteins exhibited significant changes, implicating pathways related to coagulation, oxidative stress, and acid‐base balance (Kobayashi et al. 2021). These findings suggest that exercise‐induced EVs play a multifaceted role in physiological adaptation, influencing metabolic, hemostatic, and homeostatic processes during and after high‐intensity exercise. Each of these facets may improve the metabolic responses to exercise, enhancing performance and/or the adaptive responses to exercise training and ultimately contributing to an enhanced quality of living.

Taken together, exercise‐induced changes in circulating EVs are influenced by exercise modality, intensity, sex, and metabolic state of the study subjects. But identifying the tissue of origin of these vesicles still poses a challenge, warranting a closer examination of EVs released directly from skeletal muscle tissues in response to exercise.

## 3 The Effects of Exercise on Skeletal Muscle‐Derived EVs

Although circulating EVs have been extensively characterized, these studies only provide a limited insight into skeletal muscle‐specific EV biology, especially as the skeletal muscle does not appear to be a major contributor to the circulating EV pool (Estrada et al. 2022; Guescini et al. 2015; Ismaeel et al. 2023; Warnier et al. 2022; S. Ma et al. 2023) despite its relatively large size (∼30%–40% of a person's body mass). In accord, the detection of myomiRs (miR‐1, −206, −431, and −486) at very low amounts in plasma‐derived EVs further supports the limited presence of SkM‐derived EVs in the circulation (J. Lovett et al. 2024; Ismaeel et al. 2023; Watanabe et al. 2022). This low abundance, coupled with the limited specificity of *α*‐sarcoglycan (which is often used as a skeletal muscle marker despite its expression in other tissues, such as the heart, smooth muscle, and lung), currently hinders investigation of their fate after exercise (Watanabe et al. 2022). Clarifying whether and how these EVs are distributed could provide critical insight into their contribution to the benefits of exercise and their potential role in promoting metabolic health.

### 3.1 SkM‐EVs and Their Distribution Following Exercise

A study using ex vivo models demonstrated that skeletal muscle secretes EVs and identified myofibers as their primary source by employing a dual fluorescent reporter mouse strain and spectral flow cytometry. *I* *n vivo*, approximately 5% of circulating tetraspanin‐positive EVs were traced to SkM myofibers (Estrada et al. 2022). Interestingly, SkM‐EV secretion is also determined by the tissue's metabolic activity, as oxidative muscles like the soleus produced smaller‐sized EVs in higher numbers compared to glycolytic muscles like the plantaris (Estrada et al. 2022; Kargl et al. 2023). This observation raises an interesting question regarding how EV release and distribution differ among those with primarily fast‐ or slow‐twitch muscle phenotypes. Given that rodents typically exhibit a primarily fast‐twitch muscle fiber‐type distribution (Delp and Duan 1996), there may be differences between what is observed in rodent models versus that in humans.

Gene expression analyses have further explored the extent to which SkM contributes to post‐exercise EV dynamics. EV concentrations in the plasma of healthy subjects increased during exercise, then decreased post‐exercise. A protein level increase in MVB biogenesis markers TSG101, CD81, and HSP60 was measured after exercise in plasma‐EVs. However, the protein and mRNA levels of these MVB biogenesis markers did not show a significant decrease in skeletal muscle after exercise, and thus the authors suggested that this tissue did not likely play a major role in the exercise‐induced increase in circulating EVs (Warnier et al. 2022). It is important to note that muscle samples were collected immediately before and directly after 60 min of exercise. This timing may have significantly influenced the findings, as the absence of detectable mRNA variations could be due to suboptimal measurement intervals. If additional MVB biogenesis marker proteins were synthesized and rapidly released via EVs during exercise, their transient presence might explain their lack of detection of any changes in their abundance in the sampled skeletal muscle tissue.

**FIGURE 2:**
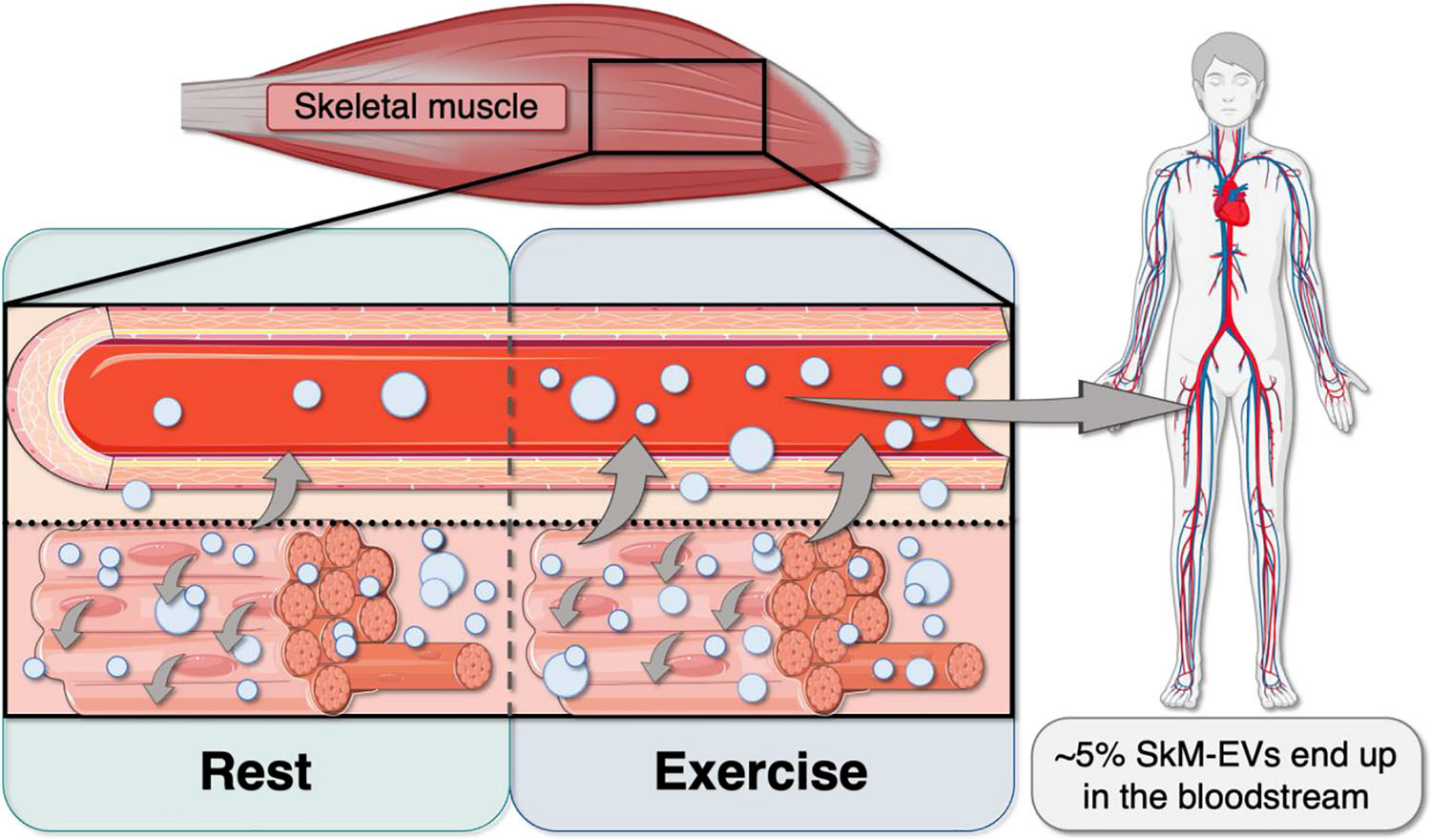
**Schematic representation of skeletal muscle‐derived extracellular vesicle release following exercise**. Exercise stimulates an increased secretion of SkM‐EVs. While most EVs remain within the interstitial space, only a smaller portion, ∼5%, enters the circulation to potentially mediate systemic effects.

To investigate whether exercise influences SkM‐EV secretion *in vivo*, Ma and colleagues used AAV vectors encoding the EV marker CD63 fused with enhanced green fluorescent protein (EGFP) with the muscle‐specific MHCK7 promoter to ensure exclusive expression in muscle cells. These MHCK7‐CD63‐EGFP‐AAV vectors were locally injected into the bilateral skeletal muscles of the mouse hindlimbs. Despite the targeted delivery into hindlimb muscles and the muscle‐specific promoter‐driven expression of the EGFP‐tagged EV marker CD63, EGFP‐positive EVs were detected in EVs enriched from serum, with levels increasing after exercise compared to baseline. Additionally, these labeled SkM‐EVs were rapidly taken up by several organs within one hour post‐injection (S. Ma et al. 2023) (Figure 2). Furthermore, this study revealed, for the first time, that SkM‐EV concentrations in serum increase following exercise, emphasizing the potential of SkM‐EVs as critical players in intercellular communication, linking skeletal muscle activity to whole‐body responses that may support systemic health and improved health span.

In contrast, other recent studies have produced more nuanced evidence, suggesting that most SkM‐EVs accumulate in the muscle microenvironment, particularly in the interstitial space, rather than entering the bloodstream at baseline or after exercise (Watanabe et al. 2022; Ismaeel et al. 2023). Notably, subtle yet significant increases in interstitial EV size have been observed following exercise, while plasma EV size distribution remained unchanged. To identify potential proteins that mark EVs released from skeletal muscle fibers, a study reported that myotube‐derived EVs contain the typical skeletal muscle proteins ATP2A1, β‐enolase, calsequestrin 2, caveolin‐3, and desmin. These proteins were detected at a different level in EVs isolated from skeletal muscle interstitium but were much less abundant or undetectable in plasma EVs. Moreover, neither of them was affected by the exercise in plasma and interstitial EVs. Of note, ATP2A1, β‐enolase, calsequestrin 2, caveolin‐3, and desmin are not exclusively expressed in skeletal muscle; they are also found in cardiac and, to a lesser extent, smooth muscle (Watanabe et al. 2022).

These findings indicate that SkM‐EVs primarily localize within the interstitium and contribute minimally to circulating EVs. Moreover, the results suggest that exercise does not significantly promote the systemic release of EVs from skeletal muscle. Instead, EVs released to the interstitial space appear to regulate local gene expression, highlighting a functional distinction from circulating EVs and a local signaling profile of SkM‐EVs (Watanabe et al. 2022). These discrepancies may reflect limitations of the models and detection approaches mentioned above, such as leaky promoter activity, vector diffusion and the inability of EV isolation methods to fully distinguish EVs from protein aggregates. The use of fluorescently tagged tetraspanins as EV reporters to track EV biogenesis and fate also raises several concerns, especially when considering that these reporters have additional functions, such as the EV marker tetraspanin CD63, which is also essential for sorting cholesterol into endosomes for storage and distribution via the exosomes (Palmulli et al. 2024). In particular, the consequences of modifying key proteins involved in EV biogenesis for both EV function and parental cell biology remain poorly understood. Moreover, overexpression of these tagged proteins could lead to alterations in EV size, cargo loading, and release, while the presence of fluorescently tagged tetraspanins on the EV surface may introduce steric hindrance, potentially impacting uptake by recipient cells. Alternative labeling approaches, such as lipophilic dyes, also present significant drawbacks, including the presence of unbound dye, aggregates or micelle formation, and nonspecific labeling of non‐EV particles. Another strategy involves the incorporation of fluorescent proteins (e.g., GFP or tdTomato) into the EV lumen through expression in the parental cell. This approach raises important questions regarding the impact of protein overexpression on cell biology, the influence of these proteins on EV size and composition and the mechanisms by which such exogenous proteins are packaged into EVs (Verweij et al. 2021; Boudna et al. 2024).

While skeletal muscle is a known source of EVs, there is still no definitive evidence that exercise directly enhances SkM‐EV secretion into the circulation. The discrepancy between interstitial EV accumulation and circulating EV levels highlights a significant gap in our understanding of SkM‐EV release. Future studies should focus on developing precise tracking methods, exploring the temporal dynamics of SkM‐EV release, and examining the role of SkM‐EVs in local versus systemic signaling. Clarifying these aspects will be critical to establishing the physiological significance of SkM‐EVs in muscle adaptation and systemic communication.

### 3.2 Exercise‐Induced Alterations in SkM‐EV Cargo

Accumulating evidence indicates that SkM‐EVs, like circulating EVs, are involved in intercellular communication, particularly by transporting miRNAs that regulate muscle physiology and metabolic pathways (F. Zhang et al. 2025). A study reported that among circulating EVs, alpha‐sarcoglycan‐positive EVs were enriched with myomiR‐206 (Guescini et al. 2015) which has been reported to modulate fat metabolism in diabetes (Wu et al. 2017). More recent research highlights differences between plasma EVs and SkM‐interstitial EVs, the latter showing higher concentrations of myomiRs (miRs‐1, −206, −431, −486). These myomiRs regulate muscle stem cell proliferation, myogenesis, and muscle regeneration by targeting the transcription factor Pax7, as well as promoting myosin heavy chain (MyHC) expression (Watanabe et al. 2022), suggesting that they primarily act within the muscle microenvironment where they accumulate.

**FIGURE 3:**
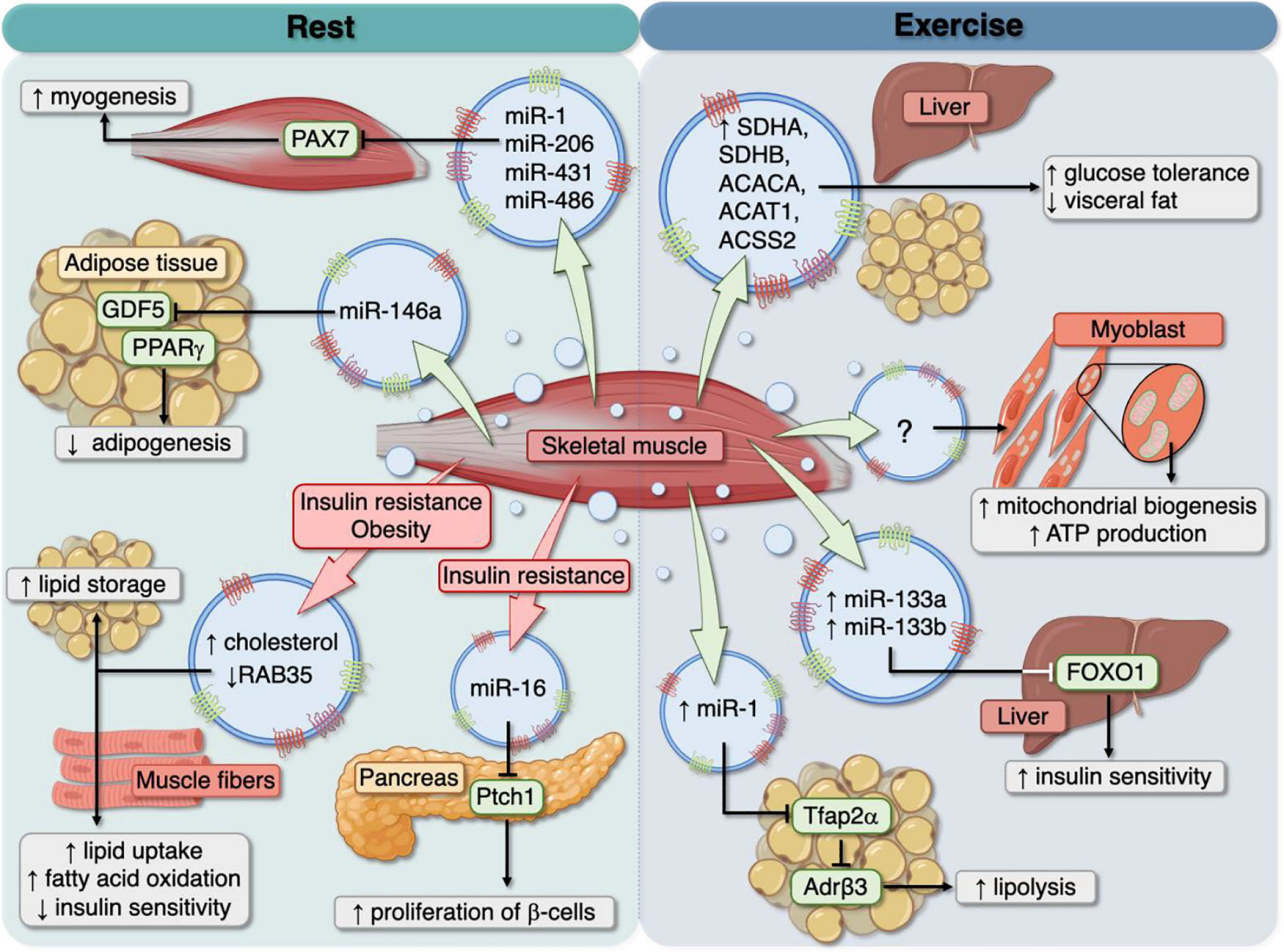
**Schematic representation of the proposed downstream effects of skeletal muscle‐derived extracellular vesicular cargo on key metabolic tissues at rest and during exercise**. SkM‐EVs carry bioactive cargo, including proteins, lipids, and microRNAs, that may influence adipose tissue, liver, pancreas and skeletal muscle tissue itself by modulating metabolic pathways. This highlights the potential role of SkM‐EVs in mediating systemic adaptations to exercise.

In partial agreement with the first studies, miR‐133a as well as miR‐133b were upregulated in SkM‐EVs following HIIT (Table 1). Of note, miR‐133a and miR‐133b target the insulin‐regulated transcription factor FoxO1, and SkM‐EVs isolated from exercised mice were enriched in miR‐133a and miR‐133b, and improved glucose tolerance when injected into sedentary mice (Castaño et al. 2020) (Table 1) (Figure 3).

**TABLE 1:** Studies investigating SkM‐EVs' microRNA cargo.

MicroRNA	EV sources	Species	Exercise treatment	Regulation	Physiological effect	Detected in plasma‐EV? (Kim et al. 2015)	Reference
miR‐1‐3p	Cultured myotubes	Human	EPS	**↑**	Promotes lipolysis as a result of enhanced catecholamine sensitivity	**+**	(Vechetti et al. 2021)
miR‐133a‐3p	Skeletal muscle	Mouse	High‐intensity interval training	**↑**	Improved glucose tolerance	**+**	(Castaño et al. 2020)
miR‐133b‐3p				**↑**		**+**	
miR‐1‐3p	Skeletal muscle	Mouse	N/A	N/A	Promote myoblast differentiation	**+**	(Watanabe et al. 2022)
miR‐206				N/A		**+**	
miR‐431				N/A		**+**	
miR‐486				N/A		**+**	
miR‐1301‐3p	Skeletal muscle	Human	Concurrent aerobic and resistance training	**↑**	Upregulation of PPARγ signaling, improvements in exercise tolerance, lipid metabolism, and mitochondrial biogenesis.	**+**	(Sullivan et al. 2022)
miR‐1307‐3p				**↑**		**+**	
miR‐146b‐5p				**↑**		**+**	
miR‐23a‐5p				**↑**		**+**	
miR‐296‐3p				**↑**		**+**	
miR‐3605‐3p				**↑**		−	
miR‐3615				**↑**		**+**	
miR‐370‐3p				**↑**		**+**	
miR‐3960				**↑**		**+**	
miR‐409‐3p				**↑**		**+**	
miR‐4326				**↑**		−	
miR‐4485‐3p				**↑**		**+**	
miR‐4485‐5p				**↑**		**+**	
miR‐4488				**↑**		**+**	
miR‐4497				**↑**		**+**	
miR‐483‐3p				**↑**		**+**	
miR‐483‐5p				**↑**		**+**	
miR‐485‐5p				**↑**		**+**	
miR‐486‐5p				**↑**		**+**	
miR‐629‐5p				**↑**		**+**	
miR‐190a‐5p				**↓**		**+**	
miR‐199a‐5p				**↓**		**+**	
miR‐199b‐5p				**↓**		**+**	
miR‐208b‐5p				**↓**		**+**	
miR‐3609				**↓**		−	
let‐7f‐2‐3p				**↓**		**+**	
miR‐101‐5p				**↓**		−	

**↑**, up‐regulation; **↓**, down‐regulation compared to controls; **+**, presence; −, absence

EPS of skeletal muscle myotubes serves as an *in vitro* model for exercise, replicating key physiological and pathological changes. It enables investigations into how different signals affect EV biogenesis and cargo. Vechetti et al. demonstrated that EPS promotes the export of miR‐1 via EVs, which are preferentially taken up by epididymal white adipose tissue (eWAT). In eWAT, miR‐1 enhances adrenergic signaling and lipolysis by targeting Tfap2α, a repressor of Adrβ3 expression (Vechetti et al. 2021) (Table 1) (Figure 3). The growing body of evidence supports the pivotal role of SkM‐EVs in exercise‐induced metabolic adaptations, particularly through miRNA‐mediated regulation of the glucose metabolism pathway.

However, SkM‐EVs influence biological processes beyond fuel metabolism, and the physiological state of the organism profoundly shapes their molecular cargo (Christiansen et al. 2025). For example, obesity and exercise have opposing effects on the miRNA profiles of SkM‐EVs. Obesity is associated with altered miRNAs that regulate pro‐inflammatory and growth‐related signaling pathways, including SERPINF1, death receptor, Gαi, Wnt/β‐catenin, PTEN, PI3K/AKT, and IGF‐1. In contrast, exercise induces anti‐inflammatory shifts in SkM‐EV cargo, modulating key pathways such as IL‐10, IL‐8, Toll‐like receptors (TLRs), and NF‐κB in both lean and obese individuals (Sullivan et al. 2022). The studies reporting SkM‐EVs' miRNA content before and after exercise in humans and experimental animals are summarised in Table 1. Aerobic and resistance exercise have also been shown to regulate genes involved in MVB biogenesis and miRNA processing. In skeletal muscle, both of these exercise modalities upregulate genes encoding proteins associated with MVB formation and the ESCRT pathway, including ALIX and clathrin. Similarly, genes involved in miRNA processing, such as Exportin‐5 and DICER, also show increased expression following exercise (Garner et al. 2020). This coordinated regulation suggests that exercise may enhance not only the biogenesis of EVs but also the packaging of miRNAs into these vesicles. EVs carry different types of RNA species that vary in abundance and proportions. These include RNAs with known functions (e.g., mRNA, miRNA and siRNA), RNA with predicted functions (e.g., vault RNA) and RNA with unknown functions, such as fragmented and degraded (methylated and uridylidated) RNA species (O'Brien et al. 2020). However, to date, the studies investigating the RNA cargo and function of exercise‐induced SkM‐EVs and their contribution to metabolic regulation have focused exclusively on miRNA. Future research should explore the specific mechanisms governing SkM‐EV biogenesis, cargo selection, and tissue‐specific uptake to unlock their therapeutic potential.

### 3.3 Downstream Effects of Skeletal Muscle‐Derived EVs on Metabolism and Glucose Homeostasis

SkM‐EVs appear to reflect the metabolic state of the tissue. EVs from leptin‐deficient mice, which exhibit insulin resistance and muscle atrophy, had altered proteomic and lipidomic profiles. These EVs promoted lipid storage in adipocytes, increased lipid uptake, fatty acid oxidation, and impaired insulin sensitivity in recipient myotubes, mirroring the phenotype of the donor muscle. This suggests a muscle‐to‐adipocyte communication pathway favoring adipose tissue expansion, in addition to muscle‐to‐muscle interaction (Jalabert et al. 2021). Of note, skeletal muscle and adipose tissue are important contributors to whole‐body energy metabolism, and their EV‐mediated communication likely facilitates their synchronized metabolic responses (Guo et al. 2023). Supporting this interorgan dialogue in a different study, SkM‐EVs inhibited preadipocyte differentiation and adipogenesis. These SkM‐EVs were highly enriched in miR‐146a‐5p, a negative regulator of peroxisome proliferator‐activated receptor γ (PPARγ) signaling, via the growth and differentiation factor 5 (GDF5) gene, affecting adipogenesis and fatty acid absorption (Qin et al. 2023) (Figure 3).

SkM‐EVs from exercised mice improved glucose tolerance and reduced visceral fat when injected into obese mice. These EVs, enriched with proteins related to metabolism and mitochondrial function, including succinate dehydrogenase A & B (SDHA & SDHB), acetyl‐CoA carboxylase (ACACA), acetyl‐CoA acetyltransferase 1 (ACAT1), acyl‐CoA synthetase short‐chain family member 2 (ACSS2), were taken up by major metabolic tissues, especially the liver and adipose tissue, improving systemic metabolic health (Yixiao Wang et al. 2023) (Figure 3). Additionally, SkM‐derived small EVs from trained mice, enriched in miR‐133a and miR‐133b, improved glucose tolerance in sedentary mice through miRNA‐mediated modulation of FoxO1, underscoring their systemic metabolic effects (Castaño et al. 2020) (Figure 3). Exercise‐induced increases in enzymes involved in carbohydrate metabolism, like lactate dehydrogenase A within EVs, align with their function in metabolic regulation (S. Ma et al. 2023), especially when considering lactate's role in skeletal muscle metabolism *in vivo*. Interestingly, *in vitro*, chronic contractile activity (CCA) (repeated electrical stimulation of myotubes) not only heightened mitochondrial biogenesis in muscle cells but also increased the release of SkM‐EVs. When myoblasts were treated with these EVs, they showed higher rates of mitochondrial biogenesis, enhanced energy production (ATP), and improved oxygen consumption in a dose‐dependent way. Together, these results suggest that SkM‐EVs can mediate pro‐metabolic effects in recipient cells and thereby transmit the effects associated with traditional exercise (Obi et al. 2025) (Figure 3).

SkM‐EVs contribute to crosstalk between skeletal muscle (SkM) and pancreatic beta‐cells as well as the liver, suggesting a potential role for these vesicles in the development of insulin resistance and T2DM (Aswad et al. 2014; Jalabert et al. 2016). *I* *n vivo*, SkM‐EVs were localized to the pancreas within 24 h post‐injection via the circulation. In this study, mice fed with a high‐palmitate diet (HPD) had a higher body weight, were glucose‐intolerant and insulin‐resistant compared with mice fed with a standard diet. *I* *n vitro*, SkM‐EVs overexpressing miR‐16 derived from HPD mice can be taken up by pancreatic beta cells, regulating Ptch1 (involved in pancreas development) and promoting beta‐cell proliferation (Figure 3). These results suggest SkM‐derived EVs mediate beta‐cell mass adaptation during insulin resistance (Jalabert et al. 2016). Given that diabetes is a leading cause of morbidity and mortality worldwide and severely reduces the health span, understanding the role of SkM‐derived EVs in beta‐cell adaptation offers promising avenues for therapeutic intervention and disease prevention.

The evidence presented supports the notion that SkM‐EVs play a multifaceted role in metabolic health, acting as mediators of intercellular communication within muscle tissue and, to a lesser extent, through systemic circulation among different organs. Despite their low abundance in plasma, SkM‐EVs carry distinct molecular cargo, including proteins, lipids, and miRNAs that influence key metabolic processes, including glucose tolerance, lipid metabolism, and inflammation regulation. Emerging evidence underscores the localized effects of SkM‐EVs within the muscle microenvironment, where they regulate myogenesis and gene expression, while also highlighting their systemic impact when released into circulation, particularly in response to exercise. The molecular composition of SkM‐EVs is altered based on physiological states such as exercise, obesity, and insulin resistance, reflecting their potential as biomarkers and therapeutic agents. The crosstalk between skeletal muscle and other organs has emerged as a key component connecting structured physical activity with positive health outcomes, and exercise represents a particularly powerful stimulus for SkM‐EV production and release. Future research should aim to further understand the mechanisms underlying SkM‐EV biogenesis and cargo selection in different physiological states to harness their full potential in improving metabolic health, combating metabolic disorders, and increasing the health span.

## 4 SkM‐EV Biogenesis and Release in Response to Exercise

EVs include the main subtypes: microvesicles (or ectosomes) and exosomes, which differ in their biogenesis, size, cargo and release pathways. While microvesicles originate from direct budding of the plasma membrane, exosomes originate from the endosomal system and are formed as intraluminal vesicles within multivesicular bodies (MVB) and secreted upon their fusion with the plasma membrane (Figure 1) (Welsh et al. 2024). Although the regulation of most EV subtypes by exercise remains poorly understood, experimental evidence supports calcium‐induced exosome secretion. The release of exosomes, a small EV species, is regulated by a calcium‐dependent mechanism, where elevated intracellular Ca^2^^+^ promotes secretion (Williams et al. 2023; Savina et al. 2003). In skeletal muscle, contractions elevate intracellular Ca^2^^+^ through the sarcoplasmic reticulum in response to neural signals. Thus, muscle contractions during exercise and the subsequent Ca^2+^ flux can enhance SkM‐EV secretion, providing a possible mechanism for contraction‐induced EV release (Savina et al. 2003). *I* *n vitro* studies exhibited mixed results regarding the effects of EPS on EV release from skeletal muscle myotubes. Short‐term stimulation, either 1 h at 14 V, 1 Hz (Bydak et al. 2022) or 8 h at 12 V, 1 Hz (Vechetti et al. 2021), did not alter EV biophysical properties (size, concentration) or exosomal marker expression. In contrast, Murata et al. demonstrated that prolonged stimulation (24 h at 10 V, 30 Hz) enhanced EV secretion and upregulated the EV biogenesis marker ALIX, an effect associated with intracellular Ca^2^^+^ peaks. Notably, 1 Hz stimulation in the same study failed to elicit these responses (Murata et al. 2023). Similarly, Obi et al. investigated the effects of CCA, where myotubes were electrically paced for 3 h per day over 4 days at 14 V, 1 Hz. While CCA did not alter EV size, it significantly increased EV secretion, accompanied by higher expression of small‐EV protein markers CD81, Tsg101, and HSP70 (Obi et al. 2025). In contrast, evidence from *ex vivo* stimulation of intact skeletal muscle remains limited. To date, only one study has directly addressed this question. Estrada et al. (2022) electrically stimulated isolated EDL muscles (30 Hz every 2 s for 10 min) under exercise‐mimicking conditions and reported no change in EV size and no increase in EV abundance in the conditioned medium (Estrada et al. 2022).

These discrepancies likely reflect fundamental differences between *in vitro* models and intact muscle preparations. Unlike cultured myotubes, whole muscle retains its three‐dimensional structure and extracellular matrix (ECM). Contraction‐induced EVs may therefore remain partially trapped within the interstitial space or ECM, limiting their diffusion into the surrounding medium and leading to an underestimation of secretion in ex vivo models.

Similarly, ultrasound stimulation, which raises intracellular Ca^2^^+^, was shown to enhance EV release from skeletal muscle cells. These EVs, enriched with miRNAs like miR‐206‐3p and miR‐378a‐3p, exhibited anti‐inflammatory properties and reduced macrophage pro‐inflammatory factors (Yamaguchi et al. 2023).

Beyond the identification of potential physiological triggers of EV release, the molecular machinery necessary for said release is only partially understood. Proteins of the Endosomal Sorting Complex Required for Transport (ESCRT), such as TSG101, ALIX, and VPS4, are linked to exosome release. However, their role in the formation of small EV precursors termed intraluminal vesicles within MVB remains incompletely understood. Additionally, Rab GTPases, including Rab27a and Rab27b, have been identified as key regulators of small EV secretion in HeLa cells (Ostrowski et al. 2010), yet it is unknown whether their function is conserved in skeletal muscle as well. Rab27a and Rab27b are small GTPases primarily involved in the trafficking and fusion of secretory vesicles to the plasma membrane, a process not typically prominent in skeletal muscle cells, whose primary function is contraction. While some studies showed low or undetectable expression of Rab27a/b in muscle (Kesari et al. 2008; Chen et al. 2002), the exact function of these GTPases in muscle tissue remains understudied compared to other cell types. Recent evidence highlights the Ca^2^^+^‐dependent Rab‐binding protein Munc13‐4 (Ca^2+^‐dependent SNAP receptor– and Rab‐binding protein) as a critical mediator of Ca^2^^+^‐dependent membrane fusion in breast carcinoma cell lines (Messenger et al. 2018). Munc13‐4 transcripts are expressed at low levels in various tissues, including skeletal muscle, suggesting a potential role in cellular processes such as vesicle trafficking and exocytosis. However, the functional role of Munc13‐4 in skeletal muscle remains uncharacterized (Koch et al. 2000).

Additionally, another factor necessary for Ca^2+^‐dependent exosome secretion is Annexin A6 (ANXA6), which is recruited to MVBs in the presence of Ca^2+^. In the HCT116 human colon carcinoma cell line, ANXA6 depletion stalls MVBs at the cell periphery, and truncated versions of ANXA6 localize to different membranes, suggesting that ANXA6 may serve to facilitate tethering MVBs to the plasma membrane, a prerequisite for the release of a small EV species (Williams et al. 2023).

Despite the advances detailed above, key gaps in our knowledge remain regarding the mechanisms underlying SkM‐EV secretion. Although calcium influx initiated by nerve signals in muscle is the more likely mechanism to trigger EV release, the effects of mechanotransduction appear to be underestimated in the release of SkM‐EVs. Piezo1, a mechanosensitive ion chanel, notably expressed in skeletal muscle, detects mechanical stimuli such as membrane stretch and shear stress, triggering calcium influx and downstream signaling pathways that could facilitate EV secretion (Lei et al. 2024; Mirzoev 2023). Interestingly, similar mechanisms have been observed in red blood cells, where mechanical stimulation and Piezo1 activation via agonists like Yoda1 induce EV release (Sangha et al. 2023). These findings might suggest that Piezo1 functions as a universal mechanosensor, mediating cellular responses to mechanical stimuli and driving EV secretion. During muscle contraction, intracellular calcium flux is driven by motor neuron activity that triggers voltage‐dependent Ca^2^^+^ release from the sarcoplasmic reticulum. Conversely, Piezo1‐mediated Ca^2^^+^ influx is activated by mechanical membrane deformation, such as shear stress, compression, or stretch, independently of neural stimuli (Coste et al. 2010; Syeda et al. 2016). Mechanical activation of Piezo1 could synergistically increase intracellular Ca^2^^+^ levels or activate parallel signaling cascades that modulate EV biogenesis or cargo sorting. However, the precise mechanisms by which Piezo1 regulates SkM‐EV release remain unclear and warrant further investigation. Elucidating these pathways may enable targeted interventions to mimic the benefits of structured physical activity in sedentary or mobility‐limited individuals, offering new approaches to extend health span and prevent age‐related functional decline. Future studies should explore the downstream signaling pathways involved and determine whether modulating Piezo1 activity can influence EV biogenesis and cargo composition.

## 5 Mechanism of Action Mediating the Effects of Exercise via SkM‐EV

Following their release, EVs interact with recipient cells to deliver their bioactive cargo (including proteins, miRNAs, lipids) and modulate the cellular function of recipient cells. This process might involve membrane docking, receptor activation, endocytosis, or direct fusion with target cells, but the molecular mechanisms regulating EV uptake and cargo transfer remain poorly understood. Importantly, EV‐cell interactions are also influenced by both the cellular origin of the EV and the recipient cell itself and likely affect the specificity of downstream responses (Van Niel et al. 2018; Welsh et al. 2024). Moreover, the characterization of their mechanisms of action is difficult to pinpoint because of the heterogeneity and complex composition of EVs, rooted in their cell type of origin, and the physiological or pathological state of said parental cells. The biodistribution, notably the ability of SkM‐EVs to reach the circulation, as well as the EV uptake by target cells and tissues add another level of complexity for which the underlying mechanisms remain largely unexplored. Nevertheless, emerging evidence suggests that exercise‐induced SkM‐EVs contribute to systemic metabolic regulation by improving glucose tolerance, modulating lipid metabolism in the liver and adipose tissue (Yixiao Wang et al. 2023), enhancing glucose homeostasis (Castaño et al. 2020; S. Ma et al. 2023), and promoting mitochondrial biogenesis (Obi et al. 2025).

These systemic effects appear to be mediated by cargo‐dependent signaling mechanisms, whereby exercise‐derived SkM‐EVs transfer miRNAs that modulate key metabolic pathways in recipient tissues. For instance, EV‐mediated delivery of miRNAs (including miR‐1, miR‐133a, and miR‐133b) targets transcriptional regulators such as Tfap2α and FoxO1, thereby influencing adrenergic signaling and lipid metabolism in adipose tissue as well as glucose homeostasis in the liver (Castaño et al. 2020; Vechetti et al. 2021) (Figure 3). In parallel, the enrichment of specific proteins within exercise‐induced SkM‐EVs, including SDHA, SDHB, ACACA, ACAT1, and ACSS2, supports pathways involved in fatty acid oxidation, carbon and pyruvate metabolism, consistent with increasing glucose tolerance and reducing lipid accumulation in recipient tissues (Yixiao Wang et al. 2023) (Figure 3). However, existing studies remain limited to a subset of identified cargo molecules. Future work will require a comprehensive characterization of SkM‐EV cargo and the pathways they regulate across target tissues to define the mechanisms underlying exercise‐induced metabolic benefits.

## 6 Challenges and Conclusions

Based on recent investigations, SkM‐EVs appear to play a role as mediators of the beneficial effects of exercise, contributing to crosstalk between skeletal muscle and other organs (Jia et al. 2025). However, significant challenges must be addressed to validate this hypothesis. One of the main limitations currently remains the ability to reliably identify the skeletal muscle origin and track the fate of these vesicles, mainly because of the lack of specific and robust skeletal muscle markers on the EVs’ surface. Moreover, both endogenous and exogenous EV labeling strategies may alter EV composition or functionality, thereby potentially biasing conclusions regarding EV uptake, biodistribution, and trafficking (Boudna et al. 2024). Consequently, identifying robust skeletal muscle‐specific markers or developing innovative EV‐tracking strategies would facilitate investigations of EV biogenesis, biodistribution and cellular uptake while minimizing bias.

A major additional limitation that remains to be addressed is the reliance on in vitro or *exvivo* models. To date, most studies have focused on EVs derived from differentiated myotube cell cultures or from skeletal muscle explants (Jia et al. 2025; Vechetti et al. 2021; Castaño et al. 2020; Watanabe et al. 2022; Sullivan et al. 2022). While these models are informative, they are not without important limitations. Myotubes differentiated from cell lines such as C2C12 cells exhibit morphological and physiological properties that differ substantially from those of mature skeletal muscle fibers. Myotubes represent immature, developing multinucleated muscle cells characterized by centrally located nuclei and an incompletely organized contractile apparatus, whereas skeletal muscle fibers (myofibers) are mature, fully functional cells with peripheral nuclei and highly organized sarcomeric structures. Moreover, skeletal muscle tissues contain varying ratios of different fiber types, determining the contractile and metabolic properties of skeletal muscle tissue by fiber‐type distribution (Hu et al. 2024; Hamaguchi et al. 2025; Cao and Warren 2025). If SkM‐EV secretion is influenced by the tissue's metabolic activity, with oxidative muscles producing smaller‐sized EVs in higher numbers compared to glycolytic muscles (Estrada et al. 2022; Kargl et al. 2023), then the in vitro ‘monoculture’ model of differentiated C2C12 myotubes likely falls short in recapitulating the complex native muscle environment comprised of different fibe types.

Conversely, studies isolating EVs from skeletal muscle explants may yield heterogeneous EV populations originating not only from muscle fibers but also from other resident cell types, such as satellite cells, fibroblasts, immune cells, endothelial cells, and even blood‐derived cells (Yizhuo Wang et al. 2025). The development of experimental systems enabling the selective isolation, culture, and electrical stimulation of contractile skeletal muscle fibers, coupled with rigorous EV isolation and functional characterization, is therefore critically needed to advance mechanistic understanding of exercise‐induced SkM‐specific EV biology.

A consideration of note is that the methodologies employed for SkM‐EV isolation vary significantly across the studies reviewed, potentially influencing the reported results. Comparative analyses of different EV isolation techniques from biofluids have demonstrated marked differences in yield, purity, and composition (Boulestreau et al. 2024; Suresh and Zhang 2025; Buschmann et al. 2018). Accordingly, SkM‐EVs collected using various techniques, including ultracentrifugation, immuno‐capture, size exclusion chromatography (SEC), and co‐precipitation, will be influenced by the inherent advantages and limitations of said enrichment techniques (Welsh et al. 2024). For instance, ultracentrifugation, the most commonly used method, allows for the recovery of a broad spectrum of EVs but may co‐isolate protein aggregates and lipoproteins. Immuno‐capture enhances specificity by targeting EV surface markers but may selectively enrich for particular subpopulations while excluding others. SEC methods provide a rapid and scalable alternative but can lead to contamination with non‐vesicular components such as chylomicrons and other lipoprotein‐rich particles. Co‐precipitation techniques facilitate high‐yield EV isolation but may introduce contaminants that affect downstream analyses. Consequently, the choice of EV enrichment approach has a direct impact on the quality and cross‐study comparability of subsequently generated omics datasets (Singh et al. 2025). The contaminating proteins co‐isolating with EVs may also confound proteomic data, affecting the identification of specific biomarkers and the definition of the functions of these proteins in signaling pathways or interorgan communication. Of note, the wide array of available proteomic instrumentation can provide varying degrees of resolution, mass accuracy and dynamic range, which can all influence EV protein cargo analysis (Dakup et al. 2025).

Similarly, miRNA profiling has been conducted using different platforms, including next‐generation sequencing (NGS), RT‐qPCR, and RT‐qPCR arrays, each impacting data interpretation. NGS provides a comprehensive and unbiased profile of EV‐miRNAs, allowing for the detection of novel sequences, but it is cost‐intensive and requires bioinformatics expertise. RT‐qPCR offers high sensitivity and specificity but is limited to known targets and requires careful normalization. RT‐qPCR arrays enable multiplex analysis of selected miRNAs but are restricted by predefined panels. These methodological differences in both EV isolation and miRNA detection could account for discrepancies in reported SkM‐EV miRNA profiles and their functional implications. Future research should aim for standardization in isolation and detection protocols to ensure comparability and reproducibility across studies.

RNA sequencing has shown that EVs carry diverse RNA biotypes, including small non‐coding RNAs (e.g., snoRNA, snRNA, Y‐RNA, tRNA), lncRNA, vault RNA, rRNA and fragments of longer transcripts, with overall RNA profiles dominated by shorter species (∼25–700 nucleotides) and variable reports on full‐length mRNAs (Dellar et al. 2022). The mechanisms governing RNA loading into EVs remain unknown: some studies argue for selective, active sorting based on RNA motifs, binding proteins and lipid interactions, while others attribute most cargo to intracellular abundance and passive loading (Dellar et al. 2022). To date, investigations of exercise effects on SkM‐EVs have focused almost exclusively on miRNAs, representing a major blind spot given the potential functional and regulatory roles of other RNA classes.

The narrow focus of studies primarily on miRNA and protein cargo of SkM‐EVs highlights a persistent lack of comprehensive characterization of EV cargo, notably overlooking not only different RNA species but also lipids and metabolites transported by these vesicles. This limits our understanding of EV‐mediated signaling and functional complexity. As a first step in addressing this gap in our knowledge, two recent studies have begun to explore the lipid cargo of SkM‐EVs, representing an important first step toward a more integrative and unbiased characterization (Jalabert et al. 2021; Hakkar et al. 2025).

Moving forward, standardization in SkM‐EV research is essential to address current challenges. Variability in EV profiles due to differences in exercise protocols, sample handling, and isolation techniques hinders cross‐study comparisons and biomarker validation. Future investigations should prioritize the comprehensive characterization of SkM‐EV cargo using advanced ‐omics approaches. Identifying specific bioactive molecules, including myo‐miRs, cytokines, and other signaling components, could elucidate how SkM‐EVs mediate exercise‐induced metabolic benefits. These discoveries have the potential to yield biomarkers predictive of exercise responsiveness and disease risk, enabling personalized strategies to promote longevity and optimize interventions that support healthy aging. Additionally, developing therapeutic strategies to mimic or enhance the molecular effects of exercise could revolutionize interventions for individuals unable to engage in sufficient physical activity, offering new avenues for combating metabolic diseases and ultimately enabling these individuals to regain their functional capacity. Finally, adopting scalable and reproducible EV isolation techniques, such as size‐exclusion chromatography (SEC), will be critical for advancing both research and clinical translation.

In summary, recent evidence shows that skeletal muscle–derived EVs can mediate exercise‐induced metabolic adaptation via the transfer of bioactive cargo that impacts glucose homeostasis, lipid metabolism, inflammation, and mitochondrial function both locally and in distant tissues. However, important questions remain regarding the contribution of SkM‑EVs to the circulating EV pool, their biodistribution, and the mechanisms regulating their release, targeting, and tissue uptake following exercise. Answering these questions will require better muscle‑specific EV markers, refined *in vivo* EV‐tracking approaches, standardized isolation methodologies, and comprehensive multi‑omics profiling of EV cargo. Addressing these challenges will unlock the potential of SkM‐EVs, paving the way for innovative, exercise‐inspired precision medicine to address metabolic disorders, frailty, and sarcopenia effectively. Ultimately, leveraging SkM‐EVs as therapeutic agents or diagnostics could transform preventive healthcare and extend the period of life spent in good health.

## Author Contributions

**Jeremy Boulestreau**: writing – original draft, writing – review and editing, visualization, conceptualization. **Scott K. Ferguson**: writing – review and editing, writing – original draft. **Luke Nelson**: writing – review and editing, writing – original draft. **Noemi Polgar**: writing – original draft, writing – review and editing, funding acquisition, project administration, supervision, conceptualization.

## Funding

This work was funded by grants from the NIH, grant numbers U54MD007601 and P20GM113134.

## Conflicts of Interest

The authors declare no conflicts of interest.

## Declaration of Generative AI and AI‐Assisted Technologies

During the preparation of this review, the authors used Grammarly in order to improve the language and readability of the manuscript. After using these tools/services, the authors reviewed and edited the content as needed and take full responsibility for the content of the published article.

## Data Availability

Data sharing is not applicable to this article as no datasets were generated or analyzed during the current study.

## References

[jex270139-bib-0001] Amosse, J. , M. Durcin , M. Malloci , et al. 2018. “Phenotyping of Circulating Extracellular Vesicles (EVs) in Obesity Identifies Large EVs as Functional Conveyors of Macrophage Migration Inhibitory Factor.” Molecular Metabolism 18: 134–142. 10.1016/j.molmet.2018.10.001. 30473096 PMC6309717

[jex270139-bib-0002] Annibalini, G. , S. Contarelli , F. Lucertini , et al. 2019. “Muscle and Systemic Molecular Responses to a Single Flywheel Based Iso‐Inertial Training Session in Resistance‐Trained Men.” Frontiers in Physiology 10: 554. 10.3389/fphys.2019.00554. 31143128 PMC6521220

[jex270139-bib-0003] Aswad, H. , A. Forterre , O. P. B. Wiklander , et al. 2014. “Exosomes Participate in the Alteration of Muscle Homeostasis During Lipid‐Induced Insulin Resistance in Mice.” Diabetologia 57, no. 10: 2155–2164. 10.1007/s00125-014-3337-2. 25073444 PMC4153976

[jex270139-bib-0004] Bei, Y. , T. Xu , D. Lv , et al. 2017. “Exercise‐Induced Circulating Extracellular Vesicles Protect Against Cardiac Ischemia–Reperfusion Injury.” Basic Research in Cardiology 112, no. 4: 38. 10.1007/s00395-017-0628-z. 28534118 PMC5748384

[jex270139-bib-0005] Boudna, M. , A. D. Campos , P. Vychytilova‐Faltejskova , T. Machackova , O. Slaby , and K. Souckova . 2024. “Strategies for Labelling of Exogenous and Endogenous Extracellular Vesicles and Their Application for in Vitro and in Vivo Functional Studies.” Cell Communication and Signaling 22, no. 1: 171. 10.1186/s12964-024-01548-3. 38461237 PMC10924393

[jex270139-bib-0006] Boulestreau, J. , L. Molina , A. Ouedraogo , et al. 2024. “Salivary Extracellular Vesicles Isolation Methods Impact the Robustness of Downstream Biomarkers Detection.” Scientific Reports 14, no. 1: 31233. 10.1038/s41598-024-82488-3. 39732788 PMC11682200

[jex270139-bib-0007] Brahmer, A. , E. Neuberger , L. Esch‐Heisser , et al. 2019. “Platelets, Endothelial Cells and Leukocytes Contribute to the Exercise‐Triggered Release of Extracellular Vesicles Into the Circulation.” Journal of Extracellular Vesicles 8, no. 1: 1615820. 10.1080/20013078.2019.1615820. 31191831 PMC6542154

[jex270139-bib-0008] Burke, B. I. , A. Ismaeel , D. E. Long , et al. 2024. “Extracellular Vesicle Transfer of miR‐1 to Adipose Tissue Modifies Lipolytic Pathways Following Resistance Exercise.” JCI Insight 9, no. 21: e182589. 10.1172/jci.insight.182589. 39316445 PMC11601556

[jex270139-bib-0009] Buschmann, D. , B. Kirchner , S. Hermann , et al. 2018. “Evaluation of Serum Extracellular Vesicle Isolation Methods for Profiling miRNAs by Next‐Generation Sequencing.” Journal of Extracellular Vesicles 7, no. 1: 1481321. 10.1080/20013078.2018.1481321. 29887978 PMC5990937

[jex270139-bib-0010] Bydak, B. , T. M. Pierdoná , S. Seif , et al. 2022. “Characterizing Extracellular Vesicles and Particles Derived From Skeletal Muscle Myoblasts and Myotubes and the Effect of Acute Contractile Activity.” Membranes 12, no. 5: 464. 10.3390/membranes12050464. 35629791 PMC9144336

[jex270139-bib-0011] Cao, T. , and C. R. Warren . 2025. “From 2D Myotube Cultures to 3D Engineered Skeletal Muscle Constructs: A Comprehensive Review of in Vitro Skeletal Muscle Models and Disease Modeling Applications.” Cells 14, no. 12: 882. 10.3390/cells14120882. 40558510 PMC12191219

[jex270139-bib-0012] Capkovic, K. L. , S. Stevenson , M. C. Johnson , J. J. Thelen , and D. D. W. Cornelison . 2008. “Neural Cell Adhesion Molecule (NCAM) Marks Adult Myogenic Cells Committed to Differentiation.” Experimental Cell Research 314, no. 7: 1553–1565. 10.1016/j.yexcr.2008.01.021. 18308302 PMC3461306

[jex270139-bib-0013] Castaño, C. , S. Kalko , A. Novials , and M. Párrizas . 2018. “Obesity‐Associated Exosomal miRNAs Modulate Glucose and Lipid Metabolism in Mice.” Proceedings of the National Academy of Sciences 115, no. 48: 12158–12163. 10.1073/pnas.1808855115. PMC6275521 30429322

[jex270139-bib-0014] Castaño, C. , M. Mirasierra , M. Vallejo , A. Novials , and M. Párrizas . 2020. “Delivery of Muscle‐Derived Exosomal miRNAs Induced by HIIT Improves Insulin Sensitivity Through Down‐Regulation of Hepatic FoxO1 in Mice.” Proceedings of the National Academy of Sciences 117, no. 48: 30335–30343. 10.1073/pnas.2016112117. PMC7720135 33199621

[jex270139-bib-0015] Chen, Y. , P. Samaraweera , T.‐T. Sun , G. Kreibich , and S. J. Orlow . 2002. “Rab27b Association With Melanosomes: Dominant Negative Mutants Disrupt Melanosomal Movement.” Journal of Investigative Dermatology 118, no. 6: 933–940. 10.1046/j.1523-1747.2002.01754.x. 12060386

[jex270139-bib-0016] Chow, L. S. , R. E. Gerszten , J. M. Taylor , et al. 2022. “Exerkines in Health, Resilience and Disease.” Nature Reviews Endocrinology 18, no. 5: 273–289. 10.1038/s41574-022-00641-2. PMC9554896 35304603

[jex270139-bib-0017] Christiansen, S. F. , K. B. F. Haug , M. Hussain , et al. 2025. “Comparative Proteomics and Micro‐RNA Analysis of Skeletal Muscle Cell Small Extracellular Vesicles—Unique Profiles in Cells From Severely Obese Individuals With Type 2 Diabetes Versus Normal Glucose Tolerance.” Frontiers in Physiology 16: 1696916. 10.3389/fphys.2025.1696916. 41347018 PMC12672307

[jex270139-bib-0018] Conkright, W. R. , M. E. Beckner , A. J. Sterczala , et al. 2022. “Resistance Exercise Differentially Alters Extracellular Vesicle Size and Subpopulation Characteristics in Healthy Men and Women: An Observational Cohort Study.” Physiological Genomics 54, no. 9: 350–359. 10.1152/physiolgenomics.00171.2021. 35816651

[jex270139-bib-0019] Coste, B. , J. Mathur , M. Schmidt , et al. 2010. “Piezo1 and Piezo2 are Essential Components of Distinct Mechanically Activated Cation Channels.” Science 330, no. 6000: 55–60. 10.1126/science.1193270. 20813920 PMC3062430

[jex270139-bib-0020] Dakup, P. P. , I. D. Ludovico , Y. You , et al. 2025. “Challenges and Opportunities in State‐of‐the‐Art Proteomics Analysis for Biomarker Development From Plasma Extracellular Vesicles.” Proteomics September 16, e70036. 10.1002/pmic.70036. 40955644 PMC12604859

[jex270139-bib-0021] Dellar, E. R. , C. Hill , G. E. Melling , D. R. F. Carter , and L. A. Baena‐Lopez .. 2022. “Unpacking Extracellular Vesicles: RNA Cargo Loading and Function.” Journal of Extracellular Biology 1, no. 5: e40. 10.1002/jex2.40. 38939528 PMC11080855

[jex270139-bib-0022] Delp, M. D. , and C. Duan . 1996. “Composition and Size of Type I, IIA, IID/X, and IIB Fibers and Citrate Synthase Activity of Rat Muscle.” Journal of Applied Physiology 80, no. 1: 261–270. 10.1152/jappl.1996.80.1.261. 8847313

[jex270139-bib-0023] Dimassi, S. , E. Karkeni , P. Laurant , Z. Tabka , J.‐F. Landrier , and C. Riva . 2018. “Microparticle miRNAs as Biomarkers of Vascular Function and Inflammation Response to Aerobic Exercise in Obesity?” Obesity (Silver Spring) 26, no. 10: 1584–1593. 10.1002/oby.22298. 30260095

[jex270139-bib-0024] D'Souza, R. F. , J. S. T. Woodhead , N. Zeng , et al. 2018. “Circulatory Exosomal miRNA Following Intense Exercise is Unrelated to Muscle and Plasma miRNA Abundances.” American Journal of Physiology‐Endocrinology and Metabolism 315, no. 4: E723–E733. 10.1152/ajpendo.00138.2018. 29969318

[jex270139-bib-0025] Du, X. , Y. Yang , C. Xu , et al. 2017. “Upregulation of miR‐181a Impairs Hepatic Glucose and Lipid Homeostasis.” Oncotarget 8, no. 53: 91362–91378. 10.18632/oncotarget.20523. 29207650 PMC5710930

[jex270139-bib-0026] Estrada, A. L. , Z. J. Valenti , G. Hehn , et al. 2022. “Extracellular Vesicle Secretion is Tissue‐Dependent Ex Vivo and Skeletal Muscle Myofiber Extracellular Vesicles Reach the Circulation in Vivo.” American Journal of Physiology. Cell Physiology 322, no. 2: C246–C259. 10.1152/ajpcell.00580.2020. 34910603 PMC8816621

[jex270139-bib-0027] Fernandez‐Sanjurjo, M. , P. Pinto‐Hernandez , A. Dávalos , et al. 2024. “Next‐Generation Sequencing Reveals That miR‐16‐5p, miR‐19a‐3p, miR‐451a, and miR‐25‐3p Cargo in Plasma Extracellular Vesicles Differentiates Sedentary Young Males From Athletes.” European Journal of Sport Science 24, no. 6: 766–776. 10.1002/ejsc.12087. 38874986 PMC11235846

[jex270139-bib-0028] Frühbeis, C. , S. Helmig , S. Tug , P. Simon , and E.‐M. Krämer‐Albers . 2015. “Physical Exercise Induces Rapid Release of Small Extracellular Vesicles Into the Circulation.” Journal of Extracellular Vesicles 4, no. 1: 28239. 10.3402/jev.v4.28239. 26142461 PMC4491306

[jex270139-bib-0029] Gaesser, G. A. , S. E. Hall , S. S. Angadi , D. C. Poole , and S. B. Racette . 2025. “Increasing the Health Span: Unique Role for Exercise.” Journal of Applied Physiology 138, no. 6: 1285–1308. 10.1152/japplphysiol.00049.2025. 40244910 PMC12182867

[jex270139-bib-0030] Garcia, N. A. , J. Moncayo‐Arlandi , P. Sepulveda , and A. Diez‐Juan . 2016. “Cardiomyocyte Exosomes Regulate Glycolytic Flux in Endothelium by Direct Transfer of GLUT Transporters and Glycolytic Enzymes.” Cardiovascular Research 109, no. 3: 397–408. 10.1093/cvr/cvv260. 26609058

[jex270139-bib-0031] Garner, R. T. , J. S. Solfest , Y. Nie , S. Kuang , J. Stout , and T. P. Gavin . 2020. “Multivesicular Body and Exosome Pathway Responses to Acute Exercise.” Experimental Physiology 105, no. 3: 511–521. 10.1113/EP088017. 31917487

[jex270139-bib-0032] Guescini, M. , B. Canonico , F. Lucertini , et al. 2015. “Muscle Releases Alpha‐Sarcoglycan Positive Extracellular Vesicles Carrying miRNAs in the Bloodstream.” PLoS ONE 10, no. 5: e0125094. 10.1371/journal.pone.0125094. 25955720 PMC4425492

[jex270139-bib-0033] Guo, L. , M. Quan , W. Pang , Y. Yin , and F. Li . 2023. “Cytokines and Exosomal miRNAs in Skeletal Muscle–Adipose Crosstalk.” Trends in Endocrinology & Metabolism 34, no. 10: 666–681. 10.1016/j.tem.2023.07.006. 37599201

[jex270139-bib-0034] Hakkar, R. , C. E. Brun , P. Leblanc , et al. 2025. “Sphingolipids in Extracellular Vesicles Released From the Skeletal Muscle Plasma Membrane Control Muscle Stem Cell Fate During Muscle Regeneration.” Journal of Extracellular Vesicles 14, no. 9: e70164. 10.1002/jev2.70164. 40984725 PMC12454923

[jex270139-bib-0035] Hamaguchi, H. , T. G. Oyama , K. Oyama , Y. Manabe , N. L. Fujii , and M. Taguchi . 2025. “Combined Stimuli of Elasticity and Microgrooves Form Aligned Myotubes That Characterize Slow Twitch Muscles.” Scientific Reports 15, no. 1: 27825. 10.1038/s41598-025-12744-7. 40781258 PMC12334659

[jex270139-bib-0036] Hu, T. , Y. Furuichi , Y. Manabe , et al. 2024. “Myokine BDNF Highly Expressed in Type I Fibers Inhibits the Differentiation of Myotubes Into Type II Fibers.” Molecular Biology Reports 51, no. 1: 1143. 10.1007/s11033-024-10044-3. 39531063 PMC11557626

[jex270139-bib-0037] Iavello, A. , V. S. L. Frech , C. Gai , M. C. Deregibus , P. J. Quesenberry , and G. Camussi . 2016. “Role of Alix in miRNA Packaging During Extracellular Vesicle Biogenesis.” International Journal of Molecular Medicine 37, no. 4: 958–966. 10.3892/ijmm.2016.2488. 26935291 PMC4790646

[jex270139-bib-0038] Ismaeel, A. , D. W. Van Pelt , Z. R. Hettinger , et al. 2023. “Extracellular Vesicle Distribution and Localization in Skeletal Muscle at Rest and Following Disuse Atrophy.” Skeletal Muscle 13, no. 1: 6. 10.1186/s13395-023-00315-1. 36895061 PMC9999658

[jex270139-bib-0039] Jalabert, A. , L. Reininger , E. Berger , et al. 2021. “Profiling of Ob/Ob Mice Skeletal Muscle Exosome‐Like Vesicles Demonstrates Combined Action of miRNAs, Proteins and Lipids to Modulate Lipid Homeostasis in Recipient Cells.” Scientific Reports 11, no. 1: 21626. 10.1038/s41598-021-00983-3. 34732797 PMC8566600

[jex270139-bib-0040] Jalabert, A. , G. Vial , C. Guay , et al. 2016. “Exosome‐Like Vesicles Released From Lipid‐Induced Insulin‐Resistant Muscles Modulate Gene Expression and Proliferation of Beta Recipient Cells in Mice.” Diabetologia 59, no. 5: 1049–1058. 10.1007/s00125-016-3882-y. 26852333

[jex270139-bib-0041] Jia, J. , L. Wang , Y. Zhou , P. Zhang , and X. Chen . 2025. “Muscle‐Derived Extracellular Vesicles Mediate Crosstalk Between Skeletal Muscle and Other Organs.” Frontiers in Physiology 15: 1501957. 10.3389/fphys.2024.1501957. 39844898 PMC11750798

[jex270139-bib-0042] Ju, J. , D. Xiao , N. Shen , et al. 2020. “miR‐150 Regulates Glucose Utilization Through Targeting GLUT4 in Insulin‐Resistant Cardiomyocytes.” Acta Biochimica et Biophysica Sinica 52, no. 10: 1111–1119. 10.1093/abbs/gmaa094. 33085741

[jex270139-bib-0043] Just, J. , Y. Yan , J. Farup , et al. 2020. “Blood Flow‐Restricted Resistance Exercise Alters the Surface Profile, miRNA Cargo and Functional Impact of Circulating Extracellular Vesicles.” Scientific Reports 10, no. 1: 5835. 10.1038/s41598-020-62456-3. 32245988 PMC7125173

[jex270139-bib-0044] Kang, M. , X. Liu , Y. Fu , and W. Timothy Garvey . 2018. “Improved Systemic Metabolism and Adipocyte Biology in miR‐150 Knockout Mice.” Metabolism: Clinical and Experimental 83: 139–148. 10.1016/j.metabol.2017.12.018. 29352962 PMC6142816

[jex270139-bib-0045] Kargl, C. K. , Z. Jia , D. A. Shera , et al. 2023. “Angiogenic Potential of Skeletal Muscle Derived Extracellular Vesicles Differs Between Oxidative and Glycolytic Muscle Tissue in Mice.” Scientific Reports 13, no. 1: 18943. 10.1038/s41598-023-45787-9. 37919323 PMC10622454

[jex270139-bib-0046] Kargl, C. K. , A. J. Sterczala , D. Santucci , et al. 2024. “Circulating Extracellular Vesicle Characteristics Differ Between Men and Women Following 12 Weeks of Concurrent Exercise Training.” Physiological Reports 12, no. 9: e16016. 10.14814/phy2.16016. 38697940 PMC11065700

[jex270139-bib-0047] Katayama, M. , E. Caria , D. Van Simaeys , et al. 2025. “Exercise Training‐Induced Extracellular miR‐136‐3p Modulates Glucose Uptake and Myogenesis Through Targeting of NRDC in Human Skeletal Muscle.” Journal of Sport and Health Science 15: 101091. 10.1016/j.jshs.2025.101091. 41046976 PMC12811471

[jex270139-bib-0048] Kawanishi, N. , T. Tominaga , and K. Suzuki . 2023. “Electrical Pulse Stimulation‐Induced Muscle Contraction Alters the microRNA and mRNA Profiles of Circulating Extracellular Vesicles in Mice.” American Journal of Physiology‐Regulatory, Integrative and Comparative Physiology 324, no. 6: R761–R771. 10.1152/ajpregu.00121.2022. 37092746

[jex270139-bib-0049] Kesari, A. , M. Fukuda , S. Knoblach , et al. 2008. “Dysferlin Deficiency Shows Compensatory Induction of Rab27A/Slp2a That May Contribute to Inflammatory Onset.” American Journal of Pathology 173, no. 5: 1476–1487. 10.2353/ajpath.2008.080098. 18832576 PMC2570137

[jex270139-bib-0103] Kim, D.‐K. , J. Lee , S. R. Kim , et al. 2014. “EVpedia: a community web portal for extracellular vesicles research.” Bioinformatics 31, no. 6: 933–939. 10.1093/bioinformatics/btu741. 25388151 PMC4375401

[jex270139-bib-0050] Kobayashi, Y. , A. Eguchi , Y. Tamai , et al. 2021. “Protein Composition of Circulating Extracellular Vesicles Immediately Changed by Particular Short Time of High‐Intensity Interval Training Exercise.” Frontiers in Physiology 12: 693007. 10.3389/fphys.2021.693007. 34276412 PMC8280769

[jex270139-bib-0051] Koch, H. , K. Hofmann , and N. Brose . 2000. “Definition of Munc13‐Homology‐Domains and Characterization of a Novel Ubiquitously Expressed Munc13 Isoform.” Biochemical Journal 349, no. Pt 1: 247–253. 10.1042/bj3490247. 10861235 PMC1221144

[jex270139-bib-0052] Lang, J. J. , S. A. Prince , K. Merucci , et al. 2024. “Cardiorespiratory Fitness is a Strong and Consistent Predictor of Morbidity and Mortality Among Adults: An Overview of Meta‐Analyses Representing Over 20.9 Million Observations From 199 Unique Cohort Studies.” British Journal of Sports Medicine 58, no. 10: 556–566. 10.1136/bjsports-2023-107849. 38599681 PMC11103301

[jex270139-bib-0053] Lei, L. , Z. Wen , M. Cao , et al. 2024. “The Emerging Role of Piezo1 in the Musculoskeletal System and Disease.” Theranostics 14, no. 10: 3963–3983. 10.7150/thno.96959. 38994033 PMC11234281

[jex270139-bib-0054] Li, K. , B. Zhao , D. Wei , et al. 2020. “miR‑146a Improves Hepatic Lipid and Glucose Metabolism by Targeting MED1.” International Journal of Molecular Medicine 45, no. 2: 543–555. 10.3892/ijmm.2019.4443. 31894315 PMC6984781

[jex270139-bib-0055] Liu, R. , C. Liu , X. He , et al. 2022. “MicroRNA‐21 Promotes Pancreatic β Cell Function Through Modulating Glucose Uptake.” Nature Communications 13, no. 1: 3545. 10.1038/s41467-022-31317-0. PMC9213410 35729232

[jex270139-bib-0056] Llorente, A. , A. Brokāne , A. Mlynska , et al. 2024. “From Sweat to Hope: The Role of Exercise‐Induced Extracellular Vesicles in Cancer Prevention and Treatment.” Journal of Extracellular Vesicles 13, no. 8: e12500. 10.1002/jev2.12500. 39183543 PMC11345496

[jex270139-bib-0057] Lovett, J. , R. S. McColl , P. Durcan , I. Vechetti , and K. H. Myburgh . 2024. “Analysis of Plasma‐Derived Small Extracellular Vesicle Characteristics and microrna Cargo Following Exercise‐Induced Skeletal Muscle Damage in Men.” Physiological Reports 12, no. 18: e70056. 10.14814/phy2.70056. 39304515 PMC11415274

[jex270139-bib-0058] Lovett, J. A. C. , P. J. Durcan , and K. H. Myburgh . 2018. “Investigation of Circulating Extracellular Vesicle MicroRNA Following Two Consecutive Bouts of Muscle‐Damaging Exercise.” Frontiers in Physiology 9: 1149. 10.3389/fphys.2018.01149. 30177888 PMC6109634

[jex270139-bib-0059] Ma, C. , J. Wang , H. Liu , et al. 2018. “Moderate Exercise Enhances Endothelial Progenitor Cell Exosomes Release and Function.” Medicine & Science in Sports & Exercise 50, no. 10: 2024–2032. 10.1249/MSS.0000000000001672. 30222687

[jex270139-bib-0060] Ma, S. , X. Xing , H. Huang , et al. 2023. “Skeletal Muscle‐Derived Extracellular Vesicles Transport Glycolytic Enzymes to Mediate Muscle‐to‐Bone Crosstalk.” Cell Metabolism 35, no. 11: 2028–2043.e7. 10.1016/j.cmet.2023.10.013. 37939660

[jex270139-bib-0061] Macpherson, R. E. K. , J. S. Huber , S. Frendo‐Cumbo , J. A. Simpson , and D. C. Wright . 2015. “Adipose Tissue Insulin Action and IL‐6 Signaling After Exercise in Obese Mice.” Medicine & Science in Sports & Exercise 47, no. 10: 2034–2042. 10.1249/MSS.0000000000000660. 25785928

[jex270139-bib-0062] Maggio, S. , B. Canonico , P. Ceccaroli , et al. 2023. “Modulation of the Circulating Extracellular Vesicles in Response to Different Exercise Regimens and Study of Their Inflammatory Effects.” International Journal of Molecular Sciences 24, no. 3: 3039. 10.3390/ijms24033039. 36769362 PMC9917742

[jex270139-bib-0063] Malvandi, A. M. , M. Faraldi , V. Sansoni , L. Gerosa , J. Jaworska , and G. Lombardi . 2024. “Circulating Myo‐miRs in Physical Exercise.” Advanced Exercise and Health Science 1, no. 2: 86–98. 10.1016/j.aehs.2024.05.005.

[jex270139-bib-0064] Mastrototaro, L. , and M. Roden . 2024. “The Effects of Extracellular Vesicles and Their Cargo on Metabolism and its Adaptation to Physical Exercise in Insulin Resistance and Type 2 Diabetes.” Proteomics 24, no. 11: 2300078. 10.1002/pmic.202300078. 37525338

[jex270139-bib-0065] Messenger, S. W. , S. S. Woo , Z. Sun , and T. F. J. Martin . 2018. “A Ca^2+^‐Stimulated Exosome Release Pathway in Cancer Cells is Regulated by Munc13‐4.” Journal of Cell Biology 217, no. 8: 2877–2890. 10.1083/jcb.201710132. 29930202 PMC6080937

[jex270139-bib-0066] Mirzoev, T. M. 2023. “The Emerging Role of Piezo1 Channels in Skeletal Muscle Physiology.” Biophysical Reviews 15, no. 5: 1171–1184. 10.1007/s12551-023-01154-6. 37975010 PMC10643716

[jex270139-bib-0067] Murata, A. , H. Akiyama , H. Honda , and K. Shimizu . 2023. “Electrical Pulse Stimulation‐Induced Tetanic Exercise Simulation Increases the Secretion of Extracellular Vesicles From C2C12 Myotubes.” Biochemical and Biophysical Research Communications 672: 177–184. 10.1016/j.bbrc.2023.06.054. 37354611

[jex270139-bib-0068] Nederveen, J. P. , G. Warnier , A. Di Carlo , M. I. Nilsson , and M. A. Tarnopolsky . 2021. “Extracellular Vesicles and Exosomes: Insights From Exercise Science.” Frontiers in Physiology 11: 604274. 10.3389/fphys.2020.604274. 33597890 PMC7882633

[jex270139-bib-0069] Nuzzo, J. L. 2024. “Sex Differences in Skeletal Muscle Fiber Types: A Meta‐Analysis.” Clinical Anatomy 37, no. 1: 81–91. 10.1002/ca.24091. 37424380

[jex270139-bib-0070] Obi, P. O. , T. F. G. Souza , B. Özerkliğ , et al. 2025. “Extracellular Vesicles Released From Skeletal Muscle Post‐Chronic Contractile Activity Increase Mitochondrial Biogenesis in Recipient Myoblasts.” Journal of Extracellular Vesicles 14, no. 4: e70045. 10.1002/jev2.70045. 40205946 PMC11982704

[jex270139-bib-0071] O'Brien, K. , K. Breyne , S. Ughetto , L. C. Laurent , and X. O. Breakefield . 2020. “RNA Delivery by Extracellular Vesicles in Mammalian Cells and its Applications.” Nature Reviews Molecular Cell Biology 21, no. 10: 585–606. 10.1038/s41580-020-0251-y. 32457507 PMC7249041

[jex270139-bib-0072] Oliveira, G. P. , W. F. Porto , C. C. Palu , et al. 2018. “Effects of Acute Aerobic Exercise on Rats Serum Extracellular Vesicles Diameter, Concentration and Small RNAs Content.” Frontiers in Physiology 9: 532. 10.3389/fphys.2018.00532. 29881354 PMC5976735

[jex270139-bib-0073] Ostrowski, M. , N. B. Carmo , S. Krumeich , et al. 2010. “Rab27a and Rab27b Control Different Steps of the Exosome Secretion Pathway.” Nature Cell Biology 12, no. 1: 19–30. 10.1038/ncb2000. 19966785

[jex270139-bib-0074] Palmulli, R. , M. Couty , M. C. Piontek , et al. 2024. “CD63 Sorts Cholesterol Into Endosomes for Storage and Distribution via Exosomes.” Nature Cell Biology 26, no. 7: 1093–1109. 10.1038/s41556-024-01432-9. 38886558

[jex270139-bib-0075] Piercy, K. L. , R. P. Troiano , R. M. Ballard , et al. 2018. “The Physical Activity Guidelines for Americans.” JAMA 320, no. 19: 2020. 10.1001/jama.2018.14854. 30418471 PMC9582631

[jex270139-bib-0076] Qin, M. , L. Xing , J. Wu , et al. 2023. “Skeletal Muscle‐Derived Exosomal miR‐146a‐5p Inhibits Adipogenesis by Mediating Muscle‐Fat Axis and Targeting GDF5‐PPARγ Signaling.” International Journal of Molecular Sciences 24, no. 5: 4561. 10.3390/ijms24054561. 36901991 PMC10003660

[jex270139-bib-0077] Rigamonti, A. E. , V. Bollati , L. Pergoli , et al. 2020. “Effects of an Acute Bout of Exercise on Circulating Extracellular Vesicles: Tissue‐, Sex‐, and BMI‐Related Differences.” International Journal of Obesity 44, no. 5: 1108–1118. 10.1038/s41366-019-0460-7. 31578459

[jex270139-bib-0078] Roos, J. , M. Dahlhaus , J.‐B. Funcke , et al. 2021. “miR‐146a Regulates Insulin Sensitivity via NPR3.” Cellular and Molecular Life Sciences 78, no. 6: 2987–3003. 10.1007/s00018-020-03699-1. 33206203 PMC8004521

[jex270139-bib-0079] Sangha, G. S. , C. M. Weber , R. M. Sapp , et al. 2023. “Mechanical Stimuli Such as Shear Stress and Piezo1 Stimulation Generate Red Blood Cell Extracellular Vesicles.” Frontiers in Physiology 14: 1246910. 10.3389/fphys.2023.1246910. 37719461 PMC10502313

[jex270139-bib-0080] Savina, A. , M. Furlán , M. Vidal , and M. I. Colombo . 2003. “Exosome Release is Regulated by a Calcium‐Dependent Mechanism in K562 Cells.” Journal of Biological Chemistry 278, no. 22: 20083–20090. 10.1074/jbc.M301642200. 12639953

[jex270139-bib-0081] Seals, D. R. , and S. Melov . 2014. “Translational Geroscience: Emphasizing Function to Achieve Optimal Longevity.” Aging 6, no. 9: 718–730. 10.18632/aging.100694. 25324468 PMC4221919

[jex270139-bib-0082] Singh, M. , P. K. Tiwari , V. Kashyap , and S. Kumar .. 2025. “Proteomics of Extracellular Vesicles: Recent Updates, Challenges and Limitations.” Proteomes 13, no. 1: 12. 10.3390/proteomes13010012. 40137841 PMC11944546

[jex270139-bib-0083] Sjøberg, K. A. , C. Frøsig , R. Kjøbsted , et al. 2017. “Exercise Increases Human Skeletal Muscle Insulin Sensitivity via Coordinated Increases in Microvascular Perfusion and Molecular Signaling.” Diabetes 66, no. 6: 1501–1510. 10.2337/db16-1327. 28292969

[jex270139-bib-0084] Sullivan, B. P. , Y. Nie , S. Evans , et al. 2022. “Obesity and Exercise Training Alter Inflammatory Pathway Skeletal Muscle Small Extracellular Vesicle microRNAs.” Experimental Physiology 107, no. 5: 462–475. 10.1113/EP090062. 35293040 PMC9323446

[jex270139-bib-0085] Suresh, P. S. , and Q. Zhang . 2025. “Comprehensive Comparison of Methods for Isolation of Extracellular Vesicles From Human Plasma.” Journal of Proteome Research 24, no. 6: 2956–2967. 10.1021/acs.jproteome.5c00149. 40356199 PMC12150312

[jex270139-bib-0086] Syeda, R. , M. N. Florendo , C. D. Cox , et al. 2016. “Piezo1 Channels are Inherently Mechanosensitive.” Cell Reports 17, no. 7: 1739–1746. 10.1016/j.celrep.2016.10.033. 27829145 PMC5129625

[jex270139-bib-0087] Trefts, E. , A. S. Williams , and D. H. Wasserman . 2015. “Exercise and the Regulation of Hepatic Metabolism.” In Progress in Molecular Biology and Translational Science, vol. 135. Elsevier. 10.1016/bs.pmbts.2015.07.010. PMC4826571 26477916

[jex270139-bib-0088] Van Niel, G. , G. D'Angelo , and G. Raposo . 2018. “Shedding Light on the Cell Biology of Extracellular Vesicles.” Nature Reviews Molecular Cell Biology 19, no. 4: 213–228. 10.1038/nrm.2017.125. 29339798

[jex270139-bib-0089] Vechetti, I. J. , B. D. Peck , Y. Wen , et al. 2021. “Mechanical Overload‐Induced Muscle‐Derived Extracellular Vesicles Promote Adipose Tissue Lipolysis.” The FASEB Journal 35, no. 6: e21644. 10.1096/fj.202100242R. 34033143 PMC8607211

[jex270139-bib-0090] Verweij, F. J. , L. Balaj , C. M. Boulanger , et al. 2021. “The Power of Imaging to Understand Extracellular Vesicle Biology in Vivo.” Nature Methods 18, no. 9: 1013–1026. 10.1038/s41592-021-01206-3. 34446922 PMC8796660

[jex270139-bib-0091] Wang, Y. , Y. Liu , S. Zhang , et al. 2023. “Exercise Improves Metabolism and Alleviates Atherosclerosis via Muscle‐Derived Extracellular Vesicles.” Aging and Disease 14, no. 3: 952. 10.14336/AD.2022.1131. 37191422 PMC10187707

[jex270139-bib-0092] Wang, Y. , P. Lou , X. Zhou , et al. 2025. “Unveiling the Functional Heterogeneity of Endogenous Tissue Extracellular Vesicles in Skeletal Muscle Through Multi‐Omics.” Chemical Engineering Journal 512: 162679. 10.1016/j.cej.2025.162679.

[jex270139-bib-0093] Warnier, G. , E. De Groote , F. A. Britto , et al. 2022. “Effects of an Acute Exercise Bout in Hypoxia on Extracellular Vesicle Release in Healthy and Prediabetic Subjects.” American Journal of Physiology‐Regulatory, Integrative and Comparative Physiology 322, no. 2: R112–R122. 10.1152/ajpregu.00220.2021. 34907783

[jex270139-bib-0094] Watanabe, S. , Y. Sudo , T. Makino , et al. 2022. “Skeletal Muscle Releases Extracellular Vesicles With Distinct Protein and microRNA Signatures That Function in the Muscle Microenvironment.” PNAS Nexus 1, no. 4: pgac173. 10.1093/pnasnexus/pgac173. 36714847 PMC9802077

[jex270139-bib-0095] Welsh, J. A. , D. C. I. Goberdhan , L. O'Driscoll , et al. 2024. “Minimal Information for Studies of Extracellular Vesicles (MISEV2023): From Basic to Advanced Approaches.” Journal of Extracellular Vesicles 13, no. 2: e12404. 10.1002/jev2.12404. 38326288 PMC10850029

[jex270139-bib-0096] Whitham, M. , B. L. Parker , M. Friedrichsen , et al. 2018. “Extracellular Vesicles Provide a Means for Tissue Crosstalk During Exercise.” Cell Metabolism 27, no. 1: 237–251.e4. 10.1016/j.cmet.2017.12.001. 29320704

[jex270139-bib-0097] Williams, J. K. , J. M. Ngo , I. M. Lehman , and R. Schekman . 2023. “Annexin A6 Mediates Calcium‐Dependent Exosome Secretion During Plasma Membrane Repair.” Elife 12: e86556. 10.7554/eLife.86556. 37204294 PMC10241516

[jex270139-bib-0098] Wu, H. , T. Zhang , F. Pan , et al. 2017. “MicroRNA‐206 Prevents Hepatosteatosis and Hyperglycemia by Facilitating Insulin Signaling and Impairing Lipogenesis.” Journal of Hepatology 66, no. 4: 816–824. 10.1016/j.jhep.2016.12.016. 28025059 PMC5568011

[jex270139-bib-0099] Xhuti, D. , M. I. Nilsson , K. Manta , M. A. Tarnopolsky , and J. P. Nederveen . 2023. “Circulating Exosome‐Like Vesicle and Skeletal Muscle microRNAs are Altered With Age and Resistance Training.” Journal of Physiology 601, no. 22: 5051–5073. 10.1113/JP282663. 36722691

[jex270139-bib-0100] Yamaguchi, A. , N. Maeshige , H. Noguchi , et al. 2023. “Pulsed Ultrasound Promotes Secretion of Anti‐Inflammatory Extracellular Vesicles From Skeletal Myotubes via Elevation of Intracellular Calcium Level.” Elife 12: RP89512. 10.7554/eLife.89512. 38054662 PMC10699803

[jex270139-bib-0101] Zhang, F. , Z. Wang , and Z. Wang . 2025. “Emerging Roles of Skeletal Muscle‐Derived Exosomal miRNAs in Metabolic Regulation: A Perspective.” Aging and Disease ahead of print, November 23 00. 10.14336/AD.2025.1331. 41296936

[jex270139-bib-0102] Zhang, X. , W. E. Kraus , J. A. Houmard , J. L. Johnson , and V. B. Kraus . 2026. “Plasma Extracellular Vesicle Signatures of Metabolic Health and Exercise Response in a Pilot Study of Older Adults.” American Journal of Physiology‐Cell Physiology 330, no. 2: C379–C389. 10.1152/ajpcell.00816.2025. 41467764 PMC12866931

